# Comparison of specialist ataxia centres with non-specialist services in terms of treatment, care, health services resource utilisation and costs in the UK using patient-reported data

**DOI:** 10.1136/bmjopen-2024-084865

**Published:** 2024-09-05

**Authors:** Julie Vallortigara, Julie Greenfield, Barry Hunt, Deborah Hoffman, Suzanne Booth, Stephen Morris, Paola Giunti

**Affiliations:** 1 Ataxia Centre, Clinical and Movement Neurosciences, UCL Queen Square Institute of Neurology, University College London, London, UK; 2 Ataxia UK, London, UK; 3 Takeda Pharmaceuticals, Cambridge, Massachusetts, USA; 4 University of Cambridge, Cambridge, UK

**Keywords:** Health policy, Neurology, Chronic Disease, HEALTH ECONOMICS, Health Services Accessibility

## Abstract

**Abstract:**

**Objectives:**

This study aims to assess the patient-reported benefits and the costs of coordinated care and multidisciplinary care at specialist ataxia centres (SACs) in the UK compared with care delivered in standard neurological clinics.

**Design:**

A patient survey was distributed between March and May 2019 to patients with ataxia or carers of patients with ataxia through the Charity Ataxia UK’s mailing list, website, magazine and social media to gather information about the diagnosis, management of the ataxias in SAC and non-specialist settings, utilisation of various healthcare services and patients’ satisfaction. We compared mean resource use for each contact type and health service costs per patient, stratifying patients by whether they were currently attending a SAC or never attended one.

**Setting:**

Secondary care including SACs and general neurology clinics.

**Participants:**

We had 277 participants in the survey, aged 16 years old and over, diagnosed with ataxia and living in the UK.

**Primary outcome measures:**

Patient experience and perception of the two healthcare services settings, patient level of satisfaction, difference in healthcare services use and costs.

**Results:**

Patients gave positive feedback about the role of SAC in understanding their condition (96.8% of SAC group), in coordinating referrals to other healthcare specialists (86.6%), and in offering opportunities to take part in research studies (85.2%). Participants who attended a SAC reported a better management of their symptoms and a more personalised care received compared with participants who never attended a SAC (p<0.001). Costs were not significantly different in between those attending a SAC and those who did not. We identified some barriers for patients in accessing the SACs, and some gaps in the care provided, for which we made some recommendations.

**Conclusions:**

This study provides useful information about ataxia patient care pathways in the UK. Overall, the results showed significantly higher patient satisfaction in SAC compared with non-SAC, at similar costs. The findings can be used to inform policy recommendations on how to improve treatment and care for people with these very rare and complex neurological diseases. Improving access to SAC for patients across the UK is one key policy recommendation of this study.

**STRENGTHS AND LIMITATIONS OF THIS STUDY**

- A strength of the study is that this is a patient-centred study based on people’s experience and feedback on the treatment and care they received.
- Given the rarity of the conditions, the relatively high number of respondents recruited using the channels of the Charity supporting people with ataxia across the UK is a strength.
- The size of the population who accessed the survey is not known; therefore, it is difficult to estimate an accurate response rate.
- The cohort is not representative of the whole population of the UK living with ataxia (age and range of ataxias).
- There were only a small number of specialist ataxia centres in the UK and these were only located in England.

## Introduction

 The ataxias included in this study are a diverse collection of rare neurodegenerative diseases that are unified by gait and balance abnormalities, appendicular incoordination and abnormalities of speech. The differential diagnoses include inherited ataxias, and some individuals who have a diagnosis of idiopathic ataxia.^1^ The conditions covered in this study are disorders where ataxia is not an epiphenomenon of another neurological condition (eg, multiple sclerosis) or due to other causes such as ataxia poststroke or developmental disorders. People with ataxia experience a lack of balance and coordination, variably associated with slurred speech, double vision, tremor and difficulty in swallowing, among other symptoms. Other neurological symptoms can complicate the cerebellar syndrome, such as sensory disturbance, cognitive impairment, spasticity, auditory and visual impairment, and bladder and bowel dysfunction. Additional symptoms outside the central nervous system may arise from cardiac complications, musculoskeletal problems and endocrinological failures. Gait and balance problems often progress to the point at which patients become wheelchair-bound or even bedridden.^2^

The ataxias are a group of conditions that are clinically and genetically heterogeneous. To date, more than 100 genetic conditions have been described.^3 4^ Epidemiological evidence is lacking but estimates suggest that there are at least 10 000 adults and 500 children with progressive ataxia in the UK.^5 6^ It is possible these figures are an underestimate however due to the challenge that the ataxia diagnosis poses to clinicians. Whereas incidence rates for the progressive ataxias collectively are not known, some specific conditions have been well characterised. For example, Friedreich’s ataxia (FRDA), the most common inherited ataxia, has an estimated incidence rate of 1:29 000 among Caucasians.^7^

A lack of awareness and understanding of these rare neurological disorders among healthcare professionals (HCPs) makes their management challenging and suggests that specialist clinical services could have an important role in diagnosis and treatment.^8^,^11^ It is important to highlight that although improving the time to reach a diagnosis and early intervention for ataxia may be challenging, these improvements may ultimately reduce complications and disabilities. There are no current treatments to halt the progression of most forms of progressive ataxia but there have been recent advances in disease-modifying and symptomatic interventions, such as holistic approaches with physiotherapy and occupational therapy (OT).^1012^,^15^ Ataxia management warrants a broad and multidisciplinary approach.^16^ Patients with this condition ideally require complex care by a multidisciplinary team (MDT), including input from numerous HCPs such as neurologists, geneticists, physiotherapists, occupational therapists, speech and language therapists, and neuropsychologists.^11^ Specialist ataxia centres (SACs) can provide the necessary coordinated care, and therefore, address the specific needs of ataxia patients. The first SAC was founded in 2005 in the UK. This service is unique as it involves a collaboration between the patient support group Ataxia UK, patients and HCPs, who focus on personalised care for patients with these rare and complex conditions.^8 9^ The Charity Ataxia UK devised criteria for accreditation of these SACs as centres of excellence. The service is continually reviewed and adapted to the patient’s needs. For example, at one of the UK SACs, in London, input is received from patients attending the service by Ataxia UK representatives who support patients after clinic visits. The results of these surveys are communicated to the HCPs who are able to implement changes to the service accordingly. The UK at the time of our study had two SACs, one in London and one in Sheffield. To date, another centre of excellence for the ataxias has been accredited in Oxford.

This study aims to assess the benefits and costs of coordinated care and multidisciplinary care at SACs in the UK compared with non-specialist settings. It was part of a larger study surveying three European countries, UK, Germany and Italy; the result of the three countries was recently published.^17 18^ This project explores the patient pathways of individuals with different progressive ataxias and studies the difference in care and the resource use and cost implications of receiving care at a SAC compared with care in non-specialist settings. Here, we focus on the data only for the UK and present more detailed information from the survey going more in-depth into care pathways, patients’ experience and satisfaction and costs. In this report, we are looking at the specificity of SAC service in terms of outcomes for patients, impact of comorbidities and complexity of their ataxia on the resource use and costs in the UK. This study also expands on preliminary feedback from patients carried out by Ataxia UK (unpublished data) showing evidence of the value of SAC in being able to deliver better management of patients with ataxia.

We ran a survey in the UK to collect information from patients about the diagnosis and the management of their ataxias. We also collected and analysed patient’s views and assessed resource use and costs. The aim of this paper is to describe the patient pathway, evaluate the patient experience and finally understand the economic cost of the two different services in the UK.

## Material and methods

We designed and conducted a cross-sectional survey to gather information on patients’ experiences of diagnosis and management of the ataxias in SACs and non-specialist services. At the time of survey distribution in the UK, there were two SACs (London and Sheffield). A third SAC in Newcastle closed 6 months before the survey was rolled out.

### Patients

The general population of interest for participation in the survey was all patients (or carers of patients, as proxy) with ataxia who were 16 years old or over and lived in the UK. This age group reflects the ages of patients seen at the SACs. As the survey was distributed by Ataxia UK, the target population were those individuals who are members of Ataxia UK (comprising people with progressive ataxias such as FRDA, inherited cerebellar ataxias, sporadic or idiopathic cerebellar ataxia). The survey did not include respondents with ataxia telangiectasia as patients living with this type of ataxia are supported by another Charity in the UK.

### Patient and public engagement

The development of survey questions was informed by previous surveys on patient preferences and priorities^19^ and from patient feedback collected by the SACs (unpublished data). The involvement of patient group representatives was important in ensuring the patient perspective was central to the research. Patient representatives were part of the team that designed the research questions to be included in the survey. In addition, further patient perspective was included via a patient and carer review panel. The recruitment of participants to take part in the study was via the mailing list of the patient support group Ataxia UK. The results of this study are being publicised to people with ataxia (including participants of this survey) via the communication channels of Ataxia UK, the patient group that distributed the survey. Dissemination will be via the Ataxia UK newsletters, website and patient events, and through publications in open-access journals, in order to make the results easily accessible to patients. These results will be presented to the International Congress for Ataxia Research in London in November 2024 to reach a global audience. The patient representatives who contributed to this survey design are included in the list of authors of this manuscript. And all the patients and carers who took part in the survey are thanked in the ‘Acknowledgement’ section.

### Survey design

The survey was designed by a working group composed of patients’ representatives (from Ataxia UK), a specialist nurse in ataxia, a neurologist expert in ataxia, a health economist, a representative from the pharmaceutical industry and experts from the Costello Medical team. In addition, further patient perspective was included via a patient and carer review panel.

After review, the final survey was constructed of 64 non-obligatory questions relating to the following topics: demographics, diagnosis, referrals, patient encounters with HCPs, ataxia symptoms experienced, treatments received and patient satisfaction. The context of the study and the medical terms used in the survey were explained at the beginning of the survey (see survey form in online supplemental appendix 1).

### Data collection

Patients with ataxia or carers of patients with ataxia who were aged 16 or over were targeted for recruitment through the Ataxia UK mailing list, as well as advertisements on the Ataxia UK website, in the Ataxia UK magazine and on social media. Members and non-members of Ataxia UK could have accessed the survey, so the size of the targeted population is unknown. Invitation emails sent to potential participants provided a patient information sheet and a weblink directly to SurveyMonkey—an online platform which hosted the survey. Patients were also asked whether they wished to receive a copy of the study results. The consent of participants was obtained online. In the UK, the consent to participate in a study without the parental consent is 16 years old. The act of submitting the survey was considered as consent to take part in the study. The survey was rolled out for a period of 3 months (March–May 2019). Survey responses were exported from SurveyMonkey and collated in Microsoft Excel, where the raw data were cleaned and analysed. The database used in Excel was anonymised.

The overall data cleaning process involved the removal of all respondents who did not answer positively to all three screening questions (questions 3–5; see online supplemental appendix 1). Where the respondent gave clear contradictory responses, the responses to those questions were removed from the analysis. Incomplete surveys were not removed from the analysis, as respondents chose to answer some questions and to skip others. The number of respondents was reported for each individual question.

### Data analyses

Responses to survey questions were stratified by the following characteristics and the distribution of eligible responses was determined for each stratification independently: attendance at a SAC, comorbidities, complexity of the disease. For the attendance at a SAC (questions 23 and 23d), people who had never been to a SAC were grouped in the ‘non-SAC’ group; people who are currently going to a centre were grouped in ‘SAC’ group. There was a third group of people who used to go to a SAC but no longer go; they were grouped in the ‘USED to SAC’ group. This group was not included in the statistical analysis because they experienced a mixed care pathway. However, the results of the survey for this group have been used to identify barriers to continue to access SACs. For comorbidities, answers to question 9 were used for that stratification. People who answered ‘none’ were put in the group with no comorbidity in addition to their ataxia. People who reported other conditions were put in the group with one or multiple comorbidities due to living with another condition or several conditions in addition to their ataxia. For complexity of the disease, answers to question 43 were used to create four groups. People who did not report any of the symptoms listed in that question were put in the group 0. People who reported between 1 and 4 symptoms associated with their ataxia were put in group 1 (low level of complexity). People who reported between 5 and 8 symptoms were put in group 2 (moderate level of complexity). People who reported between 9 and 12 symptoms were put in group 3 (high level of complexity).

In questions with responses measured on a Likert scale, the responses were grouped into two categories, where affirmative responses were considered to be either ‘very positive/positive/slightly positive’ or ‘very effective/effective/slightly effective’ or ‘strongly agree/agree/slightly agree’ and negative responses were considered to be either ‘very negative/negative/slightly negative’ or ‘very ineffective/ineffective/slightly ineffective’ or ‘strongly disagree/disagree/slightly disagree ‘, respectively. For questions 56–63, affirmative responses were considered to be ‘best it could be/very well/quite well/adequately’. Results were prepared as tabulated descriptive statistics and presented as numbers (n) and percentage (%) of total respondents per question.

The questions were treated independently, and statistical tests were performed on Excel to compare results between groups non-SAC and SAC for each question using the χ^2^ Pearson test.

### Resource use and costs

We assessed resource use and costs from the perspective of the National Health Service and personal social services, which is recommended in the UK.^20^ Participants were asked to record the number of healthcare contacts they had received in the preceding 6 months specifically to manage their ataxia. Participants were asked to record the number of contacts with the general practitioner (GP), hospital outpatient clinic visits with a neurologist (not at a SAC), SAC visits, hospital inpatient stays, accident and emergency (A&E) department visits, physiotherapy appointments, speech and language therapy appointments, OT appointments and other consultant specialist visits (eg, an ophthalmologist, an ear, nose and throat specialist, urologist, gastroenterologist and others). Figures were multiplied by two to give 12-month estimates.^21^ All respondents who reported attending the SAC were recorded as having one visit to the SAC each year; any additional visits that were reported to the SAC were included in the economic analysis as other consultant specialist visits. Unit costs for each type of contact were obtained from local providers or published sources; these include staff time and workups investigations.^22^ All costs were reported in 2021 UK£. We compared average resource use for each contact type, and costs, over a 12-month period, stratifying patients by whether they were currently attending a SAC or had never attended a SAC. We then repeated this analysis, additionally stratifying by the presence of comorbidities (no/yes) and the number of symptoms experienced as a result of ataxia (0, 1–4, 5–8 and 9–12 symptoms). We tested for significant differences in mean resource use and costs between the SAC/non-SAC groups using unadjusted and adjusted ordinary least squares regression analysis, the latter controlling for age, sex, plus the number of symptoms experienced as a result of ataxia and whether or not the patient had comorbidities, as appropriate. We also tested for significant differences in mean contacts and costs between subgroups of patients group by the presence of comorbidities and the number of symptoms experienced separately for non-SAC and SAC groups using adjusted ordinary least squares regression analysis.

### Patient travel

As part of the survey, we also asked respondents to record the main mode of transport they used to travel to the specialist centre and travel time from home to the centre.

All analyses for resource use and costs were performed using Stata V.15 software.^23^

## Results

### Cohort demographics

In total, there were 277 respondents to the survey (table 1). Respondents were predominantly patients (N=234, 85.7%). There were 52.6% (N=142) female vs 47.4% (N=128) male and most respondents were in the age range 60–80 (N=140, 51.9%) years of age. With regard to the diagnosis, 114 (42.7%) respondents reported idiopathic cerebellar ataxia (CA), 78 (29.2%) inherited CA, 38 (14.2%) with other types of ataxia, 27 (10.1%) with FRDA and 10 (3.8%) with unknown type. The location of residence of respondents was asked and showed the geographical spread of participants. The most represented geographical regions were South East of England (13.7%; excluding London which was considered as a separate region), East of England (12.2%), South West of England (11.8%) and the East Midlands (10.7%) (online supplemental figure 1). When we looked at the participants in each region and stratified by SAC attendance, we noted that regions closer to an active SAC at the time the survey was rolled out, for example, London, Yorkshire and West Midlands had a high proportion of people attending a SAC vs people in non-specialist clinics (online supplemental figure 2). On the other hand, areas far away from either of the two centres (Northern Ireland, Scotland, Wales and South West of England) had a higher proportion of people in non-SAC group versus SAC group.

**Table 1:** Survey respondent demographics

Respondent type (n=273)	Patient	234 (85.7%)
	On behalf of the patient	39 (14.3%)
Age distribution (n=270)	16–29	12 (4.4%)
	30–59	106 (39.3%)
	60–80	140 (51.9%)
	80+	12 (4.4%)
Sex (n=270)	Female	142 (52.6%)
Diagnosis (n=267)	MaleFRDA	128 (47.4%)27 (10.1%)
	Inherited CA	78 (29.2%)
	Idiopathic CA	114 (42.7%)
	Other types	38 (14.2%)
	Not known	10 (3.8%)
SAC groups based on attendance (n=248)	Non-SAC	128 (51.6%)
	SAC	72 (29%)
	Used to SAC	48 (19.4%)

After data cleaning, there were 277 respondents to the survey.

Other types: episodic ataxia, gluten ataxia, hereditary spastic paraplegia, auto-immune ataxias, and sensory ataxia.

SAC groups: non-SAC=people who have never been to a SAC; SAC=people currently attending a SAC; UUsed to SAC=people who used to visit a SAC and no longer go.

CAcerebellar ataxiaFRDAFriedreich’s ataxiaSACspecialist ataxia centre

### Reaching a diagnosis

Respondents were asked how long ago they received their ataxia diagnosis. A majority reported the maximum length of time available on the survey that is, more than 5 years ago (N=146, 62.1%), followed by between 2 and 5 years ago (N=55, 23.4%), between 1 and 2 years (N=19, 8.1%), between 6 months and 1 year (N=9, 3.8%) and finally up to 6 months ago (N=6, 2.6%) (online supplemental table 1). At the time of this diagnosis, 222 respondents (94.8%) said their ataxia had an impact on their life to some degree by restricting their activities: 92 (39.9%) with ataxia caused occasional problems, 77 (32.9%) with ataxia caused frequent problems, 53 (22.6%) with ataxia caused constant problems (online supplemental table 2).

**Table 2:** HCP who referred participants to a SAC

Attendance to SAC N (%)	GP	Hospital neurologist	Other primary HCP	Other	Unsure	Total
SAC	25 (36.8)	4 (4.4)	33 (48.5)	7 (10.3)	0 (0)	68 (100)
Used to SAC	17 (36.2)	3 (6.4)	21 (44.7)	5 (10.6)	1 (2.1)	47 (100)
Unsure	2 (28.6)	0 (0)	4 (57.1)	1 (14.3)	0 (0)	7 (100)
Total	44 (36.1)	6 (4.9)	58 (47.5)	13 (10.7)	1 (0.8)	122 (100)

Others: local neurologists, private neurologist, neurologist at SAC through research study, Ataxia UK information -led participants to ask to be referred, Eear, Nnose and Tthroat consultant.

Primary care professional: someone who provides medical care outside of a hospital, for example, a (GP), nurse practitioner, pharmacist, health visitor, midwife, dentist or optician. So here, for other primary care healthcare professional, it would be any professional except the GP.

GPgeneral practitionerHCPhealthcare professionalSACspecialist ataxia centre

Respondents were asked which HCP gave this initial diagnosis of ataxia. A large majority reported that the neurologist was the professional giving them the diagnosis: 199 respondents (85.4%), followed by 13 receiving it from the geneticist (5.6%). Other answers included diagnosis from the GP (N=6, 2.6%), physiotherapist (N=1, 0.4%) and other HCPs (radiographer, audiologist, paediatrician, consultant rehabilitation neurologist; N=10, 4.3%). Respondents were also asked about the time spent between the first symptom experienced and the first referral to see a neurologist. 30% of the respondents (N=71) reported being referred at the time they first sought medical advice for their first symptom of ataxia, followed by 16.4% (N=38) who reported a referral time up to 6 months. The rest of the cohort is split between the groups between 6 months to 1 year (N+31, 13.4%), between 1 and 2 years (N=25, 10.8%), between 2 and 5 years (N=27, 11.6%) and finally more than 5 years (N=23, 9.9%).

### Attendance at a SAC

Based on the attendance at SAC versus standard neurology clinic (see the ‘Material and methods’ section), participants are categorised into three groups named as follows: never been to SAC named non-SAC (N=128, 51.6%); currently attending SAC named SAC (N=72, 29%); used to go to SAC named Used to SAC (N=48, 19.4%) (table 1).

When we looked at the comorbidity by attendance to SAC, the groups SAC and Non-SAC showed similar proportion of people with no comorbidities, and people with one or multiple comorbidities: 46.9% (N=60) of people in non-SAC group have ataxia only (no comorbidities) vs 48.9% (N=35) of people in SAC group; 44.5% (N=57) of people in non-SAC have one or multiple others conditions aside of ataxia vs 43.1% (N=31) for the people in SAC (online supplemental table 3).

When we looked at comparing the complexity of the ataxia between the groups SAC and non-SAC, we found no difference for the categories 0, 1 or 2, however, for category 3, the group non-SAC has a higher proportion of participants compared with the group SAC (online supplemental table 4; p=0.01). When we looked at the means of the number of symptoms related to ataxia rather than categories, there was no difference between the SAC group and non-SAC group in terms of complexity of the ataxia reported by participants (p=0.86).

### Patient pathway

#### Primary contact

Respondents were asked who they consider to be their primary contact for their ataxia. For respondents who currently go to SAC, 85.7% (N=60) consider the neurologist at the SAC to be their first point of contact, whereas respondents who have never been to SAC consider either their local neurologist (45%, N=45) or their GPs (36%, N=36) to be their first point of contact (online supplemental table 5).

#### Access to SAC

When we asked participants who referred them to the SAC, for those who have been to a SAC, we added similar responses from the groups SAC and used to SAC with a split between a referral made by the local neurologist (48.5% (N=33) and 44.7% (N=21), respectively) and made by the GP (36.8% (N=25) and 36.2% (N17), respectively) (table 2). A majority of the participants have been a patient at a SAC for 2 years and more (online supplemental table 6).

The reasons why people ceased receiving care at the SAC were investigated with a question. As mentioned in the ‘Material and methods’ section, one SAC in Newcastle closed 6 months before the roll out of this survey. As a result, among the 47 respondents to this question, 12 (25.5%) reported that they ceased going to a SAC because the centre where they were seen closed down and they have not been referred to another one yet. Problems with travelling and transport to the centre were also reported by 12 people (25.5%) of this group. The other answers included not referred again (N=7; 14.9%), did not find it useful (N=6; 12.8%) and other reasons (N=5; 10.6%), equal care locally (N=2; 4.3%).

For the people who have never been to a SAC, when we asked the reasons, 29 (16.6%) reported that distance was the cause of not attending a SAC. Answers in the free text boxes also mentioned travelling being an issue in attending a SAC. The other more frequent response with free text boxes (13.7%, N=24) was the lack of awareness of such centres and the lack of awareness about how to be referred there for treatment and care. The rest of the answers were: current level of care sufficient (N=18; 10.3%), asked to be referred to a SAC but was refused by my doctor (N=6; 3.4%) and did not wish to be referred (N=2; 1.1%).

#### Access to an MDT

Respondents were asked if they ever attended a multidisciplinary clinic. MDTs were defined in the question as teams including more than one of the following people: physiotherapists, occupational therapists, orthotics or speech and language therapists. Out of 230 participants, 87 (37.8%) reported attending an MDT; among them, 36 (50.7%) are in the SAC group, 30 (28.8%) are in the non-SAC group and 20 (42.6%) used to go to a SAC (table 3). Attendance at an MDT clinic is different between the groups SAC and Non-SAC (p<0.001). Overall half of the respondents in SAC group went to an MDT (50.7%, N=36)) versus nearly one-third of respondents in non-SAC (28.8%, N=30).

**Table 3:** Attendance to a multidisciplinary team clinic results are stratified by SAC attendance

SAC groups N (%)	Attended an MDT clinic	Never attended an MDT clinic	Unsure	Total
Non-SAC	30 (28.8)*	68 (65.4)	6 (5.8)	104 (100)
SAC	36 (50.7)*	31 (43.7)	4 (5.6)	71 (100)
Used to SAC	20 (42.6)	22 (46.8)	5 (10.6)	47 (100)
Unsure	1 (12.5)	7 (87.5)	0 (0)	8 (100)
Total	87 (37.8%)	128 (55.7%)	15 (6.5%)	230 (100%)

* p<0.001.

MDTmultidisciplinary teamSACspecialist ataxia centre

### Patient satisfaction

#### MDT service

Respondents were asked how effective the MDT care received was. Out of 84 people, 74 reported positive feedback (88.1%) about the MDT care being effective. The majority of the subjects in the three groups, non-SAC (N=25), SAC (N=33) and used to SAC (N=16) reported positive feedback about the MDT (83.3%, 94.3% and 84.2%, respectively).

#### SAC service

In a series of questions on the patient experience of the various healthcare services, respondents were asked their opinion on specific aspects of the management of their ataxia. People from both the SAC and used to SAC groups reported positive feedback on the following statements about the specialists they saw at the SAC: (statement 1) they understood how to manage my ataxia (96.8% (N=61) and 81.6% (N=31) were positive respectively); (statement 2) they understood the symptoms of my ataxia (96.6% (N=63) and 89.7% (N=35) were positive, respectively); (statement 3) they understood the treatments available for my ataxia (93.1% (N=54) and 71.4% (N=25) were positive, respectively (table 4).

**Table 4:** Feedback of participants on the SAC service they visited

Group N (%)	SAC		Used to SAC		Total	
Feedback	Positive	Negative	Positive	Negative	Positive	Negative
HCP understood how to manage my ataxia	61 (96.8)	2 (3.2)	31 (81.6)	7 (18.4)	92	9
Total	63 (100)		38 (100)		101	
HCP understood the symptoms of my ataxia	63 (96.6)	2 (3.1)	35 (89.7)	4 (10.3)	98	6
Total	65 (100)		39 (100)		104	
HCP understood the treatments available for my ataxia	54 (93.1)	4 (6.9)	25 (71.4)	10 (28.6)	79	14
Total	58 (100)		35 (100)		93	
Coordination of referrals to specialists	58 (86.6)	9 (13.4)	26 (61.9)	16 (38.1)	84	25
Total	67 (100)		42 (100)		109	
Offers to participate in research	46 (85.2)	8 (14.8)	24 (60)	16 (40)	70	24
Total	54 (100)		40 (100)		94	
Help with securing parking badges	20 (62.5)	12 (37.5)	19 (65.5)	10 (34.5)	39	22
Total	32 (100)		29 (100)		61	
Help with benefits	12 (40)	18 (60)	13 (40.4)	19 (59.6)	25	37
Total	30 (100)		32 (100)		62	
Liaising with social workers	6 (25)	18 (75)	8 (28.6)	20 (71.4)	14	38
Total	24 (100)		28 (100)		52	

HCP, healthcare professionalSACspecialist ataxia centre

We asked feedback about SAC service on specific items of the patient experience while being seen in a SAC. A majority of respondents who went to a SAC, either currently or used to go, reported positive feedback about the coordination of referrals to specialists (86.6% (N=58) and 61.9% (N=26), respectively); the offers to participate in research (85.2% (N=46) and 60% (N=24), respectively); the help with securing a disabled person parking badge (62.5% (N=20) and 65.5% (N=19), respectively) (table 4). On the other hand, respondents from either SAC or used to SAC group reported negative feedback about the help to get some welfare benefits (60% (N=18) and 59.6% (N=19), respectively); the liaison with social workers (75% (N=18) and 71.4% (N=20), respectively) (table 4).

#### Patient experience of other healthcare services

Respondents were asked a series of questions on their experience visiting primary, secondary care as well as A&E, sharing their opinion on the same three statements written above (see section on SAC service) for the HCP they saw, apart from the A&E service where the statements were different. People from the three groups SAC, non-SAC and used to SAC, reported similar and mixed feedback between positive and negative, on the three statements about HCPs they saw in primary care (online supplemental table 7): for the statement 1, 47.3% (N=95) gave positive feedback; for the statement 2, 52.8% (N=105) gave positive feedback; for the statement 3, 41% (N=81) with positive feedback. For the feedback about the HCPs in secondary care: respondents from the three SAC attendance groups reported primarily positive feedback for the statement 1 (63.2%, N=127) and 2 (62.2%, N=122), whereas they reported primarily negative feedback for the statement 3 (51.7%, N=93). There were differences in the feedback given by the SAC group compared with non-SAC group: for statements 1 and 2, a higher proportion of SAC patients was positive compared with non-SAC patients (p=0.01). There was no difference in the feedback given by these two groups for the statement 3. Regarding the respondent’s visit to A&E, people from the three SAC attendance groups gave primarily negative feedback about the following three statements: (statement 1) healthcare workers in A&E understood how to manage my ataxia, 82.3% (N=79) gave negative feedback; (statement 2) healthcare workers in A&E understood the symptoms of my ataxia, 81.8% (N=81) with negative feedback; (statement 3) healthcare workers in A&E understood how my ataxia might affect the treatment provided, 79.8% (N=71) (online supplemental table 7). There were differences in the feedback given by the SAC group compared with non-SAC group: for the three statements, a higher proportion of SAC patients was negative compared with non-SAC patients (p<0.001). There were a couple of follow-up questions about the experience of respondents when visiting A&E. When people were asked if the care received during the time spent in A&E could have been better, the groups SAC and used to SAC gave a similar response with 84.6% (N=11) and 85% (N=17), respectively positive feedback, whereas the group non-SAC gave a mixed response with 58.7% (N=27) positive feedback (online supplemental table 7). The difference in feedback between the SAC and non-SAC group for this question was statistically significant (p<0.001). Respondents were asked their opinion on having a card with key information on their condition to use when visiting A&E; people in the three groups were positive about this idea (online supplemental table 8).

#### Patient satisfaction with symptoms management and care received

We asked participants’ feedback on the overall management of the symptoms they experienced with their ataxia. The feedback given by the groups SAC and non-SAC is statistically different. The feedback given by people in SAC group is significantly more positive than negative: 77% (N=48) vs 23% (N=14) negative (χ^2^ test; p<0.001) (online supplemental table 9). The negative feedback given by people in non-SAC group is significantly higher than the negative feedback given by people in SAC group: 45% (N=47) vs 23% (N=14) (χ^2^ test; p<0.001).

Participants were asked their feelings about the care they received being adapted to their needs (online supplemental table 9). The feedback given by the groups SAC and non-SAC is statistically different. The people in the SAC group gave more positive feedback than negative: 75% (N=48) vs 25% (N=16) negative feedback (χ^2^ test; p<0.001). There is a difference in the negative feedback given between SAC group and non-SAC group: more negative feedback for non-SAC with 48% (N=51) people vs 25% (N=16) people in SAC (χ^2^ test; p<0.001).

### Resource use and costs

The most common contacts for SAC patients were physiotherapy visits (mean 3.1 visits per patient per year) followed by other consultant specialist visits (3.0) and GP visits (1.9) (table 5). For non-SAC patients, the most common types of contact were with the GP (2.3), then the physiotherapist (1.6) and then the standard neurology clinic (1.5). Other than for the SAC visits themselves, the differences in the numbers of contacts for the different types of health service use between the SAC and non-SAC groups were non-significant. The exception to this was the number of other visits to specialists (other than neurologists), for which there were higher mean contacts for patients in the SAC group compared with the non-SAC group (p<0.01). The mean total cost per patient over a 1-year period was £1650 for non-SAC patients and £1311 for SAC patients (p=0.59).

**Table 5:** Healthcare contacts over a 1-year period for non-SAC and SAC patients

Healthcare contacts	Patients who reported never attending a SAC				Patients who reported attending a SAC currently				Unit cost	P value*	P value†
	N	Mean	SD	Median	N	Mean	SD	Median			
Specialist centre visits	109	0	0	0	72	1	0	1	167	<0.01	<0.01
General practitioner visits	108	2.3	3.5	0	58	1.9	3.3	0	36	0.40	0.51
Neurologist outpatient visits	115	1.5	2.9	0	59	1.3	2.6	0	167	0.54	0.24
Inpatient stays	110	0.3	1.0	0	58	0.1	0.4	0	3627	0.20	0.17
Accident and emergency visits	112	0.3	1.0	0	60	0.3	1.0	0	166	0.65	0.82
Physiotherapy visits	109	1.6	3.2	0	64	3.1	4.5	0	55	0.01	0.11
Speech and language therapy visits	113	0.7	1.8	0	60	0.3	0.7	0	157	0.06	0.23
Occupational health therapy visits	113	1.3	3.1	0	63	1.5	3.0	0	102	0.81	0.98
Other consultant specialist visits	109	0.7	2.1	0	59	3.0	3.3	1	101	<0.01	<0.01
Total cost	84	1650	3789	375	51	1311	1670	803		0.55	0.59

* Test for significant differences in mean values between non-SAC and SAC groups (unadjusted).

† Test for significant differences in mean values between non-SAC and SAC groups (adjusted for age, sex, number of symptoms and the number of comorbidities).

SAC, specialist ataxia centre; N, number of participants who responded to that question

When we stratified our analyses by the presence of comorbidities, these trends remained the same. We found evidence of significantly higher physiotherapist visits and other consultant specialist visits in the SAC group compared with the non-SAC group among people with no comorbidities (p=0.02 and p<0.01, respectively); for people with comorbidities, only the number of visits to other consultant specialists was significantly different between SAC and non-SAC, SAC group reporting higher number of these visits (p<0.01) (online supplemental table 10). The mean total cost per patient over a 1-year period was £1257 for non-SAC patients with no comorbidities and £1046 for SAC patients with no comorbidities (p=0.62), and £1824 for non-SAC patients with comorbidities and £1686 for SAC patients with comorbidities (p=0.64). Among patients who did not attend a SAC, there were no differences in the mean number of contacts for any type of resource use, or for costs; the same was true for patients in the SAC group.

We also stratified our analyses by the number of symptoms as a measure of complexity, and the results of resources and costings are summarised in online supplemental table 11. The only differences between SAC and non-SAC groups were found for the two categories out of four. For the group with 1–4 symptoms, SAC patients had more visits to other consultant specialists (2.5 vs 0.6 mean visit per year, p<0.01); non-SAC patients reported more speech and language visits compared with SAC group (0.8 vs 0, p=0.05). The mean total cost per patient was £439 for non-SAC patients and £755 for SAC patients (p<0.01). In the group of patients with 5–8 symptoms, a higher number of appointments to other consultant specialists was reported by SAC group compared with non-SAC (3.5 vs 0.7, p<0.01). Overall, among patients who did not attend a SAC the numbers of inpatient stays (p=0.01), A&E visits (p=0.02) and occupational health therapy visits (p=0.04), and total costs (p=0.02), varied by the number of symptoms. Among patients who did attend a SAC the numbers of GP visits (p=0.04), and inpatient stays (p=0.03), and total costs (p<0.01), varied by the number of symptoms. Generally, values were higher in the subgroups with 1–4 and 5–8 symptoms, which were also the groups with the highest number of respondents.

### Patient travel

The modal transport time to travel to the primary ataxia treatment centre was 1–2 hours in the SAC group (58%) and <1 hour in the non-SAC group (63%) (online supplemental table 12). Although there were only two SACs in England at the time this study was undertaken, for most patients in our survey attending a SAC, travel time to the centre was 2 hours or less suggesting that referrals to SACs were geographically clustered within a 50 mile radius and there may be inequality in accessing a specialist centre for patients in England and other countries in the UK with ataxia.

## Discussion

In our previous publications^17 18^ describing the results of the survey in the three countries, we reported a significantly higher level of patient satisfaction regarding the management of the conditions and their individual needs at the SACs in the UK (77% positive feedback in SAC vs 55% positive feedback in non-SAC group (p<0.001) compared with 94% vs 88% in Germany, and 74% vs 62.5% in Italy). We, therefore, wanted to explore further the UK set of data and gain a better understanding of the care pathway, the specific role of the SAC and provide new policy recommendations in the UK. We have used data collected from a survey where the design was patient rather than HCPs, aiming to capture ataxia patients’ views, experience and feedback.

Overall, we have a better understanding of the ataxia patient care pathway and the role that the SACs play in that care. People who attend a SAC gave positive feedback about the service and reported a better management of their symptoms and more personalised care received compared with people who did not attend a SAC. Some barriers and gaps were identified in accessing a SAC and in the care provided for which we make some recommendations based on these results.

This survey consists of a cohort of patients who have mostly been living with ataxia for years, experiencing impact of the condition on their daily activities right from the time of their diagnosis. Indeed, the majority of respondents are relatively old, with over half of the cohort being in the age range 60–80 years old (51.9%), and disease duration was over 5 years for a majority of respondents (62.1%). This study describes the experience of living with the condition for decades, with progression of symptoms slowly decreasing the quality of life.

A high proportion of people (46.5%) did not have a definite specific diagnosis. Progressive ataxias are frequently very challenging to diagnose, and even with the use of whole-genome sequencing many cases remain undiagnosed.^16^ Encouragingly, recent advances in molecular genetics have enabled the diagnosis of many more cases with a genetic cause.^24^ Autoimmunity may also be important (but under-recognised) in causing some progressive cerebellar ataxias.^25^

It is crucial for people living with ataxia to see a neurologist, as this is the HCP who diagnosed most of the participants and would know about the condition and the management. We noted that a majority of participants had to wait to see a neurologist, with a delay from 6 months to over 5 years in referral after their first symptoms occurred. This represents a concern, and we think there is a need to improve the referral rate time so more people can access a neurologist sooner.

The value of seeing a neurologist with expertise and experience in ataxia is key, in order to provide personalised care and specific treatment and management for the symptoms. In our cohort, only 29% attend a SAC, which is a concern. The challenge to access a SAC was previously reported by a survey run by the Charity Ataxia UK, where 46% of people reported difficulties in accessing a SAC and about a third attending.^19^ SACs are known as the places where people can see a clinical team of experts in ataxia, discuss their symptoms and their needs, receive medical advice and specific interventions to manage their conditions.^8^,^1016^ Patients reported that the neurologist at the SAC is the point of contact for patients with ataxia once they start attending the clinic, whereas for people not attending a SAC, their first point of contact is either the local neurologist or the GP, HCPs who would not normally have the same level of expertise in ataxia. An interesting finding of our study was the difference in feedback given by participants about their visit to various HCPs and their experiences, whether they were SAC patients or not. It seems that people who currently go to SAC have a different perception and expectation of the healthcare services they visit, for example, there was more positive feedback about secondary care services, and more negative feedback about their visit to A&E. It may be that the difference of perception of SAC patients is because of a better knowledge of their condition and a better understanding of what can be offered for the management of their condition by visiting the specialist clinical team at the ataxia centre. These patients’ report regarding the A&E services is not surprising as ataxias are a group of very rare conditions.

Among the people who have been referred to a SAC, a proportion has stopped attending the service for various reasons. The closure of the Newcastle ataxia centre had a significant impact on access to SAC, leaving some patients with no referral to another service, and no other local specialist service available. The challenges of travelling to a SAC were highlighted, with potentially increased difficulties travelling to one of the remaining two centres. The travel time issue was mentioned as a barrier for people to attend a SAC, with only London and Sheffield centres running at the time of the survey, illustrated by the longer time spent travelling to a SAC compared with visiting a local neurology clinic. This suggests inequality in accessing a specialist centre and highlights the need for more centres in the UK for the care of people with ataxia. Since this study took place, another centre has been accredited in Oxford.^26^ Moreover, exhaustion and fatigue are known to be a significant burden for people with ataxia,^19^ so having specialised care closer to their home would be extremely beneficial as it seems the location plays a big part in the ability to access a centre. This is particularly the case for more remote parts of the UK, located far from the current centres, such as Scotland and Northern Ireland. We did not see a difference in comorbidities nor complexity of the disease in our cohort between people going to SAC and those going to non-specialised services, so it seems these are not factors determining the access and attendance at a SAC.

It is very important for patients to see an MDT for the management of their ataxia and the coordination of their care. The ataxias are complex conditions with a diverse set of symptoms requiring the expertise and input of various HC specialists.^9 16 19^ Similar to the access to SAC, a minority of participants attended an MDT clinic across the whole cohort. Although the access for people in SAC to an MDT was greater than those in non-SAC group, this proportion was only half of the SAC group. For those who attended such clinics, the feedback given was extremely positive in terms of the MDT care being effective for the patients. This shows the need to improve access to MDT clinics for people with ataxia in both SAC and non-specialist settings in order to make a difference in the management of the condition for more people in the UK.

Within our cohort, a majority of the people who attend a SAC have been a patient there for at least 2 years, putting them in a good position to give feedback on the service. SAC service was beneficial to these people in terms of obtaining referrals to other specialists, opportunities to take part in research, but also practical help such as obtaining a disabled parking badge. In particular, the role of the SAC team is crucial in between visits of the patients, to follow up on the care plan, liaise with the GP and local HCPs involved in people’s care. Patients who attended a SAC receive specialist ataxia nurse contacts when needed. This facilitates clinical management and the possibility of additional liaisons with the GP. Indeed, people visiting SACs reported more visits to physiotherapists and more appointments to other specialists. Overall, these referrals can make a difference by managing the condition better, addressing treatable symptoms effectively with an improvement of their daily life activities. The statistically significant difference in satisfaction with the symptoms management in the UK for people attending SACs was not seen in the two other countries (Germany and Italy). This high level of satisfaction for the UK SACs could be due to the unique involvement of the patients and patient group Ataxia UK in the development and ongoing shaping of the service with the clinical teams. This has been a novel model involving accreditation and monitoring of the ataxia centres by Ataxia UK. Aspects of the SAC service that could be improved from the participants’ perspective are around helping people to access benefits and liaising with social workers. Financial difficulties and lack of access to social services can have a significant impact on quality of life.^19^ Improving these aspects of the service could potentially facilitate a package of care that is better adapted to their needs. To tackle that gap, someone within the SAC, but also in the charity Ataxia UK could take on that role, or maybe create a new role as a care manager.

The feedback of participants about their visits to different HC services from primary, secondary and tertiary care was quite clear in terms of the level of understanding of their conditions and symptom management: the more specialised the service was, the better the patient experience. This is in accordance with the previous survey done by Ataxia UK.^19^ Moreover, the overall symptoms management and adapted care received were better in SAC compared with non-specialist clinics (positive feedback for symptom management and adapted care is 77% and 75% in SAC group vs 55% and 52% in non-SAC group, respectively). This emphasises again the importance for people with ataxia to be referred to a SAC so they can receive specialist input.

Largely our cost analysis found little evidence of difference in costs between people with ataxia who were treated at a SAC and those who were not. This was true across all patients, and also when we analysed subgroups of patients by the presence of comorbidities and the number of symptoms they were affected by. The only exception to this was in the subgroup of patients with 1–4 symptoms, where those treated at a SAC incurred significantly higher costs. This could be due to the fact that these people were at the early stage of their condition, and therefore, the need for more HCP input. Across all patients, and in several of the subgroups mean costs were lower among the patients treated at a SAC. However, the variation in costs was large and so the mean differences were not statistically significant. This could be because the sample sizes were small, especially in the analyses by subgroups. Combining the results of the cost analysis with those of the rest of the study, the findings seem to suggest that management of ataxia at a SAC can lead to better management of symptoms and improved patient satisfaction but without an appreciable difference in treatment costs. This point might be borne in mind by planners when considering increasing the provision of specialist services for ataxia.

For other healthcare services outside the SAC service, there is a need for increased awareness regarding the management of the complex ataxias to ensure patients are referred to the adapted service for their condition and their needs. When accessing an A&E service, a lack of understanding regarding the management of ataxia was reported in the survey. This represents an unmet need that should be addressed so people with these conditions can receive adapted treatment and care also in such services.

### Limitations of the study

The survey was publicised through various channels of Ataxia UK including the website and social media; therefore, non-members could have accessed the survey. In that context, it is very difficult to get an estimation of the size of the targeted population, and so the response rate is not accessible. However, we would like to stress the fact that given the rarity of the conditions, the relatively high number of respondents recruited using the channels of the Charity supporting people with ataxia across the UK is positive. The age range of our respondent cohort was somehow unusual for this kind of conditions (a majority were 60–80 years). This leads to the possibility that patients have more comorbidities than a younger population. We did not ask about the mobility status of participants, which may have helped to link results to the severity of the impairments, however, we asked about comorbidities. A majority of respondents were affected by their ataxia at the time of the first diagnosis they received. We did not ask in this survey how their daily activities are affected now. We do not have data regarding the role of the SAC in the diagnostic pathway for these rare diseases because the aim of our project was mainly focused on the management of the ataxias. Other reports in the literature focus on the delay in having a specific diagnosis in rare diseases.^27^ Participants were not asked how they got referred to an MDT to know better the pathway or the place where they saw an MDT (SAC or outside a SAC). However, as more people attended an MDT clinic from the SAC versus the non-SAC group, we can only assume that SAC facilitates referrals to see an MDT. The wording in the survey regarding visits to a SAC (question 21 of the survey, online supplemental appendix 1) could have been misunderstood because respondents were asked to report the number of visits to the SAC, and this may have been interpreted by respondents to mean the number of visits to the hospital where the SAC was, irrespective of the type of health professional seen. We know that according to routine practice in the UK people with ataxia who attended a SAC are invited to visit the centre once per year. Therefore, all respondents who reported attending the SAC were known to have one visit to the SAC each year and any additional visits that were reported were included in the economic analysis as other consultant specialist visits. Finally, when doing the analysis looking at factors such as a number of symptoms associated with ataxia, some subgroups were limited in size number.

A large survey was done in 2016 and published by Rare Disease UK with more than 1200 people affected by rare diseases participating, revealing challenges and unmet needs such as lack of communication between services, long travel to multiple clinics, missing a care coordinator or advisor and lack of awareness about the existence of a specialist centre.^27^ Our results are in line with the results from that study with the participants to our survey highlighting the barriers and needs of people living with ataxia to receive better, personalised care in order to improve management of their condition.

Since that large survey, the department of health and social care published the UK Rare Disease Framework in 2021^28^ which highlighted the four major challenges faced by the Rare Disease community: getting the correct diagnosis, awareness of Rare Diseases among HCPs, coordination of care and access to specialist care and treatments.

## Conclusion

We have conducted this survey focusing on the management of the ataxias as an example of a rare disease. We have seen from the pathways that there are some challenges in getting patients referred to a SAC. The results from the project highlight that there was no cost difference, but there were better outcomes for patients and better management in SAC compared with standard neurology clinics. Therefore, we recommend referral to SAC for patients with these rare conditions. There is also a need to improve access to MDT clinics for people with ataxia, in order to make a difference in the management of the condition to a larger number of people in the UK. In addition, looking at the geography of people living in the UK who participated in this survey, it seems that the ones likely to attend SAC are those living in the Midlands and South of England and London, near one of the two centres. There is a possibility to establish more centres to cover other regions (West country of England, Wales, Scotland, Northern Ireland) and also for existing centres to provide local support with telemedicine. In our opinion, the unique set-up of the specialist service that includes patients’ feedback allows ongoing development of the service, always in keeping with the patient’s needs that is unique in the UK. We believe that this set-up should be extended to other complex neurological diseases, rare and non-rare. Although the ataxias are a group of rare diseases, the model of care could also be easily transferred to more common neurodegenerative conditions like Parkinson’s disease or even beyond neurodegeneration to more complex conditions like rheumatic diseases.

## supplementary material

10.1136/bmjopen-2024-084865 online supplemental file 1

## Data Availability

Data are available on reasonable request.
