# Supplementary material for: Comparison of specialist ataxia centres with non-specialist services in terms of treatment, care, health services resource utilisation and costs in the UK using patient-reported data
Source: BMJ Open. 2024 Sep 5;14(9):e084865. doi: 10.1136/bmjopen-2024-084865 (PMC11381710; doi:10.1136/bmjopen-2024-084865)
Supplement: online supplemental file 1 [file bmjopen-14-9-s001.pdf]

Supplementary Figure 1: Geographical region where participants live

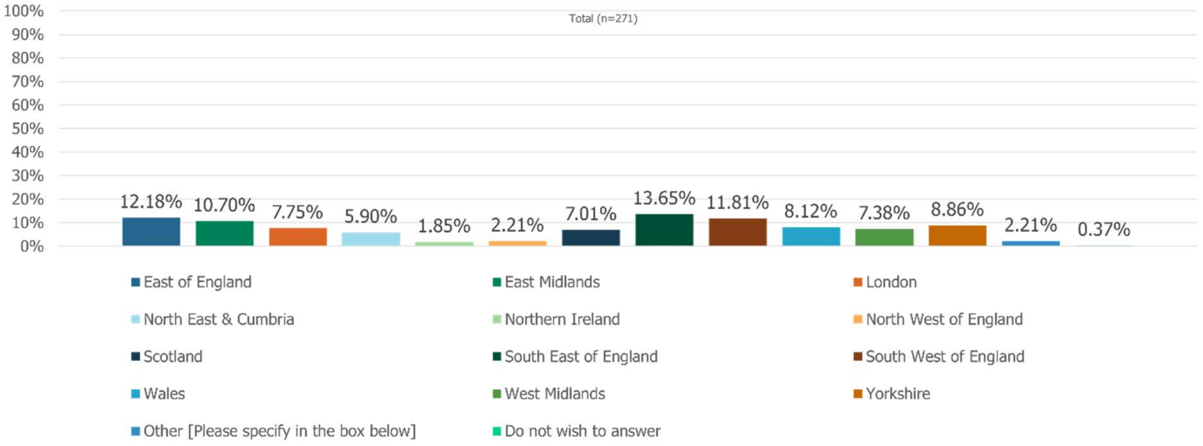

Supplementary Figure 2: Geographical region where participants live stratified by SAC attendance

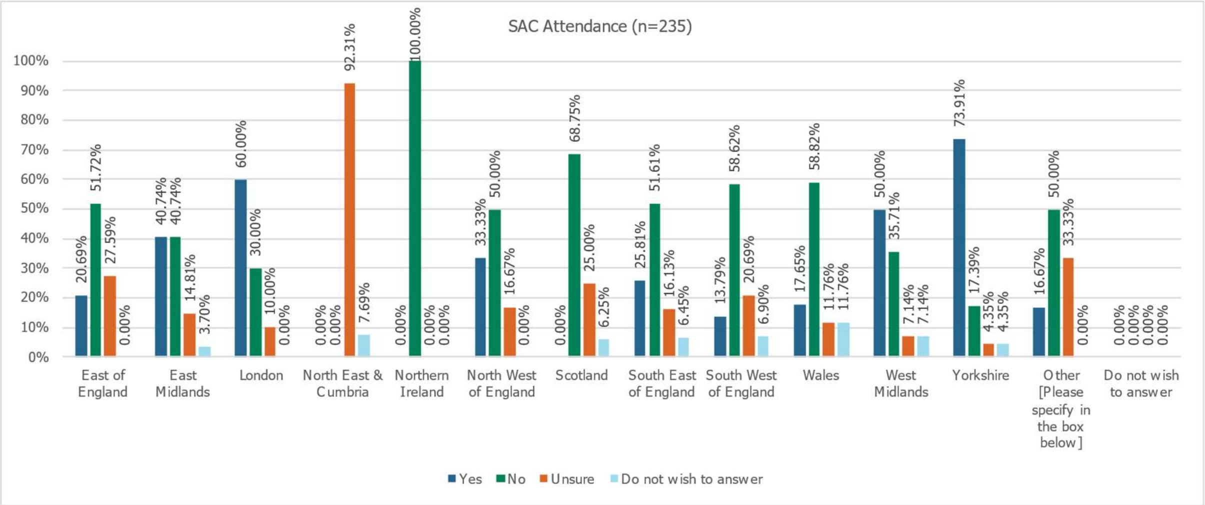

Supplementary Table 1: How long-ago participants received their first diagnosis of ataxia

Timeframe	Up to six months ago	Between six months and one year ago	Between one and two years ago	Between two and five years ago	More than five years ago	Unsure	Total
Answers N (%)	6 (2.6)	9 (3.8)	19 (8.1)	55 (23.4)	146 (62.1)	0 (0)	253 (100)

Supplementary Table 2: Impact of ataxia on participants lives at the time of first diagnosis

Level of impact	My ataxia did not affect me	My ataxia caused occasional problems	My ataxia caused frequent problems restricting my activities	My ataxia caused constant problems restricting me most of all or all of the time	Unsure	Total
Answers N (%)	9 (3.9)	92 (39.3)	77 (32.9)	55 (22.6)	3 (1.3)	234 (100)

Supplementary Table 3: Co-morbidity by attendance to SAC

Groups N (%)	No co-morbidity	One or more co-morbidity	Unsure	Total
Non-SAC	60 (46.9)	57 (44.5)	11 (8.6)	128 (100)
SAC	35 (48.6)	31 (43.1)	6 (8.3)	72 (100)
Used to SAC	29 (60.4)	18 (37.5)	1 (2.1)	48 (100)
Total	124 (50)	106 (42.7)	18 (7.3)	248 (100)

Supplementary Table 4: Number of symptoms experienced as a result of ataxia by attendance to SAC

Groups N (%)	Level 0	Level 1	Level 2	Level 3	Missing	Total
Non- SAC	10 (7.8)	46 (35.9)	38 (29.7)	17 (13.3) **	17 (13.3)	128 (100)
SAC	3 (4.2)	28 (39.4)	29 (40.9)	3 (4.2) **	8 (11.3)	71 (100)
Used to SAC	3 (6.25)	19 (39.6)	13 (27.1)	7 (14.6)	6 (12.5)	48 (100)
Unsure	0 (0)	6 (33.35)	4 (22.2)	2 (11.1)	6 (33.35)	18 (100)
Total	16 (6)	99 (37.4)	84 (31.7)	29 (10.9)	37 (14)	265 (100)

List of symptoms: pain, heart problems, gastroenterological problems, sexual dysfunction, swallowing, hearing problems, eye problems, sleep disturbances, depression, other mental health issues, fatigue.

Categories: 0= 0 symptoms, 1 = 1-4 symptoms, 2 = 5-8 symptoms, 3 = 9-12 symptoms. ** p=0.01

Supplementary Table 5: Primary HCP contact of participants for their ataxia

HCP<br>N (%)	GP	Neurologist (not at a SAC)	Neurologist at SAC	Other	Unsure	Total
Non- SAC	36 (36)	45 (45)	4 (4)	4 (4)	11 (11)	100 (44.6)
SAC	1 (1.4)	6 (8.6)	60 (85.7)	2 (2.9)	1 (1.4)	70 (31.3)
Used to SAC	7 (15.9)	18 (40.9)	11 (25)	2 (4.6)	6 (13.6)	44 (19.6)
Unsure	2 (20)	5 (50)	2 (20)	0 (0)	1 (10)	10 (4.5)
Total	46 (20.5)	74 (33)	77 (34.4)	8 (3.6)	19 (8.5)	224 (100)

Other: specialist neuro nurse, rehabilitation consultant, ataxia support group on Facebook, nobody, neurologists, Hospice neurological support worker, nurse specialist or secretary, neurophysiotherapist, specialist neurological nurse at local hospice

Supplementary Table 6: How long-ago participants were referred to SAC

Time<br>N (%)	Up to six months ago	Between six months and one year	Between one and two years	Between two and five years	More than five years ago	Unsure	Total
SAC	4 (5.6)	7 (9.9)	13 (18.3)	21 (29.6)	25 (35.2)	1 (1.4)	71 (100)
Used to SAC	2 (4.1)	1 (2.1)	8 (16.7)	14 (29.2)	22 (45.8)	1 (2.1)	48 (100)
Unsure	2 (28.6)	1 (14.3)	2 (28.5)	1 (14.3)	1 (14.3)	0 (0)	7 (100)
Total	8 (6.3)	9 (7.1)	23 (18.3)	36 (28.6)	48 (38.1)	2 (1.6)	126 (100)

Supplementary Table 7: Feedback of participants on the health care services they visited.

Group N (%)	Non- SAC		SAC		Used to SAC		Total	
Feedback	Positive	Negative	Positive	Negative	positive	Negative	Positive	Negative

Primary care HCP understood how to manage my ataxia	44 (45.4%)	53 (54.6%)	32 (53.3%)	28 (46.7%)	19 (43.2%)	25 (56.8%)	95 (47.3%)	106 (52.7%)
Total	97 (100)		60 (100)		44 (100)		201 (100)	
Primary care HCP understood the symptoms of my ataxia	50 (52.1%)	46 (47.9)	31 (51.7)	29 (48.3)	24 (55.8)	19 (44.2)	105 (52.8)	94 (45.2)
Total	96 (100)		65 (100)		39 (100)		199 (100)	
Primary care HCP understood the treatments available for my ataxia	38 (40.0%)	57 (60.0%)	26 (44.1%)	33 (55.9%)	17 (39.5%)	26 (60.5%)	81 (41%)	116 (59%)
Total	95 (100)		59 (100)		43 (100)		197 (100)	
Secondary care HCP understood how to manage my ataxia	56 (58.3%) **	40 (41.7%) **	42 (68.9%) **	19 (31.1%) **	29 (65.9%)	15 (34.1%)	127 (63.2%)	74 (36.8%)
Total	106 (100)		61 (100)		44 (100)		201 (100)	
Secondary care HCP understood the symptoms of my ataxia	54 (58.1) **	39 (41.9) **	42 (70) **	18 (30) **	26 (60.5)	17 (39.5)	122 (62.2)	74 (37.8)
Total	93 (100)		60 (100)		43 (100)		196 (100)	
Secondary care HCP understood the treatments available for my ataxia	43 (47.8%)	47 (52.2%)	26 (50.0%)	26 (50.0%)	18 (47.4%)	20 (52.6%)	87 (48.3%)	93 (51.7%)
Total	90 (100)		52 (100)		38 (100)		180 (100)	
HCP in A&E understood how to manage my ataxia	14 (25.9)	40 (74.1)	2 (10.5)	17 (89.5)	1 (4.3)	22 (95.7)	17 (17.7)	79 (82.3)
Total	64 (100)		19 (100)		23 (100)		96 (100)	
HCP in A&E understood the			5 (23.8)		2 (8.3)			

symptoms of my ataxia	11 (20.4)	43 (79.6)		16 (76.2)		22 (91.7)	18 (18.2)	81 (81.8)
Total	54 (100)		21 (100)		24 (100)		99 (100)	
HCP in A&E understood how my ataxia might affect the treatment provided to me	15 (30.6) ***	34 (69.4) ***	0 (0) ***	16 (100) ***	3 (12.5)	21 (87.5)	18 (20.2)	71 (79.8)
Total	49 (100)		16 (100)		24 (100)		89 (100)	
The care I received in A&E could have been better	27 (58.7) ***	19 (41.3) ***	11 (84.6) ***	2 (15.4) ***	17 (85)	3 (15)	55 (69.6)	24 (30.4)
Total	46 (100)		13 (100)		20 (100)		79 (100)	

Primary care healthcare professionals (HCP): GP, physiotherapist, occupational therapist; secondary care healthcare professionals (HCP): neurologist, other consultants at my local hospital; A&E: accident and emergency

** p=0.01; *** p<0.001

Supplementary Table 8: Opinion of patients about having a card with key information about their condition for use in A&E

Feedback N (%)	A great idea	A good idea	Not a bad idea	Not a good idea	A bad idea	A terrible idea	Total
Non- SAC	52 (57.1)	38 (41.8)	0 (0)	0 (0)	1 (1.1)	0 (0)	91 (100)
SAC	34 (57.6)	23 (38.6)	0 (0)	2 (3.4)	0 (0)	0 (0)	59 (100)
Used to SAC	21 (48.8)	21 (48.8)	0 (0)	0 (0)	0 (0)	1 (2.3)	43 (100)
Unsure	3 950)	2 (33.3)	0 (0)	0 (0)	0 (0)	1 (16.7)	6 (100)
Total	110 (55.3)	84 (42.2)	0 (0)	2 (1)	1 (0.5)	2 (1)	199 (100)

A&E: accident and emergency

Supplementary Table 9: Feedback of participants on symptoms management (a) and care received being adapted to people's needs (b).
Overall feedback on symptoms management (a) and care received being adapted to people's needs (b)

(a)

Feedback on symptom management N (%)	Number of respondents in Non-SAC	Number of respondents in SAC
Positive	58 (55)	48 (77) ***
Negative	47 (45) ***	14 (23) ***
Total respondents	105 (100)	62 (100)

Positive feedback means here that people feel their symptoms are well managed (question 62, Appendix 1).

(b)

Feedback on care received N (%)	Number of respondents in Non-SAC	Number of respondents in SAC
Positive	56 (52)	48 (75) ***
Negative	51 (48) ***	16 (25) ***
Total respondents	107 (100)	64 (100)

Positive feedback means here that people feel their care reflects their need (question 63, Appendix 1).

Supplementary Table 10. Health care contacts over a one-year period for non-SAC and SAC patients by presence of comorbidities

	Patients who reported never attending a SAC				Patients who reported attending a SAC currently					
**Health care contacts**	**N**	**Mean**	**Std. Dev.**	**Median**	**N**	**Mean**	**Std. Dev.**	**Median**	**P-value†**	**P-value‡**
**No comorbidities**										
Specialist centre visits	52	0	0	0	35	1	0	1	<0.01	<0.01
General Practitioner visits	49	2.0	3.2	0	29	1.3	2.4	0	0.32	0.51
Neurologist outpatient visits	53	1.2	2.0	0	29	1.4	3.1	0	0.72	0.09
Inpatient stays	50	0.1	0.5	0	29	0.1	0.4	0	0.62	0.79
Accident & Emergency visits	51	0.4	1.0	0	30	0.2	1.1	0	0.53	0.53
Physiotherapy visits	50	1.5	3.1	0	31	4.1	5.4	0	<0.01	0.02
Speech and language therapy visits	51	0.7	1.8	0	30	0.3	0.7	0	0.21	0.49
Occupational health therapy visits	52	1.2	2.7	0	30	1.7	3.6	0	0.54	0.94
Other consultant specialist visits	50	0.6	2.2	0	30	2.8	3.2	1	<0.01	<0.01
Total cost	41	1257	2954	334	25	1046	1114	704	0.73	0.62
**One or more comorbidity**										
Specialist centre visits	48	0	0	0	31	1	0	1	<0.01	<0.01
General Practitioner visits	48	2.5	3.9	0	25	2.4	4.0	0	0.95	0.85
Neurologist outpatient visits	52	1.7	3.8	0	26	1.1	2.2	0	0.45	0.81
Inpatient stays	49	0.4	1.2	0	25	0.2	0.6	0	0.34	0.17
Accident & Emergency visits	50	0.2	0.7	0	26	0.4	1.0	0	0.45	0.36
Physiotherapy visits	48	1.6	3.1	0	28	2.0	3.1	0	0.68	0.90

Speech and language therapy visits	51	0.4	1.1	0	26	0.2	0.7	0	0.39	0.44
Occupational health therapy visits	50	1.1	2.9	0	29	1.2	2.4	0	0.80	0.88
Other consultant specialist visits	48	0.9	2.3	0	25	3.4	3.7	2	<0.01	<0.01
Total cost	34	1824	4100	441	22	1686	2201	921	0.89	0.64
**P-value**§										
General Practitioner visits	0.41				0.09					
Neurologist outpatient visits	0.81				0.93					
Inpatient stays	0.29				0.89					
Accident & Emergency visits	0.23				0.95					
Physiotherapy visits	0.87				0.12					
Speech and language therapy visits	0.19				0.75					
Occupational health therapy visits	0.71				0.33					
Other consultant specialist visits	0.50				0.17					
Total cost	0.53				0.19					

† Test for significant differences in mean values between non-SAC and SAC groups (unadjusted)

‡ Test for significant differences in mean values between non-SAC and SAC groups (adjusted for age, sex and number of symptoms)

§ Test for significant differences in mean values by comorbidities separately for non-SAC and SAC groups (adjusted for age, sex and number of symptoms)

SAC, specialist ataxia centre; N, number of participants who responded to that question.

Supplementary Table 11. Health care contacts over a one-year period for non-SAC and SAC patients by number of symptoms experienced as a result of ataxia

	**Patients who reported never attending a SAC**				**Patients who reported attending a SAC currently**					
**Health care contacts**	**N**	**Mean**	**Std. Dev.**	**Median**	**N**	**Mean**	**Std. Dev.**	**Median**	**P-value†**	**P-value‡**
**No symptoms**										
Specialist centre visits	7	0	0	0	3	1	0	1	<0.01	<0.01
General Practitioner visits	9	2.9	4.0	0	3	0	0	0	0.26	0.89
Neurologist outpatient visits	10	3.2	7.4	0	3	0	0	0	0.49	0.31
Inpatient stays	6	0	0	0	3	0	0	0	.	.
Accident & Emergency visits	8	0	0	0	3	0	0	0	.	.
Physiotherapy visits	8	0.8	2.1	0	3	0	0	0	0.57	0.54
Speech and language therapy visits	8	0.3	0.7	0	3	0	0	0	0.57	0.54
Occupational health therapy visits	9	0.2	0.7	0	3	0	0	0	0.59	0.50
Other consultant specialist visits	7	0	0	0	3	0.7	0.6	1	0.01	0.36
Total cost	4	173	225	107	3	234	58	268	0.67	0.83
**1-4 symptoms**										
Specialist centre visits	40	0	0	0	28	1	0	1	<0.01	<0.01
General Practitioner visits	36	1.2	2.8	0	21	0.8	1.7	0	0.56	0.80
Neurologist outpatient visits	40	1	1.3	0	21	0.6	1.1	0	0.20	0.56
Inpatient stays	40	0	0	0	21	0	0	0	.	.

Accident & Emergency visits	39	0.1	0.3	0	22	0.3	0.9	0	0.18	0.21
Physiotherapy visits	37	1.0	2.3	0	24	2.7	4.2	0	0.06	0.08
Speech and language therapy visits	39	0.8	1.9	0	22	0	0	0	0.06	0.05
Occupational health therapy visits	39	0.36	1.0	0	23	0.8	1.3	0	0.16	0.09
Other consultant specialist visits	38	0.6	2.1	0	22	2.5	3.1	1	<0.01	<0.01
Total cost	31	439	767	110	20	755	499	636	0.11	<0.01
**5-8 symptoms**										
Specialist centre visits	34	0	0	0	30	1	0	1	<0.01	<0.01
General Practitioner visits	33	2.9	3.8	2	26	3.2	4.0	2	0.81	0.97
Neurologist outpatient visits	34	1.9	3.0	2	26	1.5	2.5	0	0.58	0.25
Inpatient stays	34	0.7	1.5	0	25	0.2	0.6	0	0.08	0.08
Accident & Emergency visits	34	0.6	1.3	0	26	0.3	1.2	0	0.39	0.46
Physiotherapy visits	34	2.8	4.4	0	28	3.6	4.8	2	0.52	0.60
Speech and language therapy visits	35	0.5	1.2	0	26	0.5	0.9	0	0.93	0.85
Occupational health therapy visits	34	2.2	4.2	0	27	1.8	3.4	0	0.65	0.65
Other consultant specialist visits	35	0.7	2.2	0	26	3.5	3.2	3	<0.01	<0.01
Total cost	25	3204	5269	892	21	1523	1742	1029	0.17	0.15
**9-12 symptoms**										
Specialist centre visits	14	0	0	0	3	1	0	1	<0.01	<0.01
General Practitioner visits	15	2.8	4.0	2	3	0	0	0	0.25	0.38

Neurologist outpatient visits	16	1.1	1.6	0	3	0	0	0	0.26	0.25
Inpatient stays	15	0.1	0.5	0	3	0.7	1.2	0	0.20	0.11
Accident & Emergency visits	16	0.4	0.8	0	3	0.7	1.2	0	0.60	0.82
Physiotherapy visits	16	1.0	1.5	0	3	1.3	2.3	0	0.74	0.61
Speech and language therapy visits	16	0.9	2.6	0	3	0	0	0	0.58	0.64
Occupational health therapy visits	16	1.6	3.0	0	3	1.3	1.2	0	0.87	0.60
Other consultant specialist visits	14	2.0	3.2	0	2	1.5	2.1	1	0.84	0.68
Total cost	11	1713	3339	344	2	4213	5006	4213	0.38	0.33
**P-value**§										
General Practitioner visits	0.11				0.04					
Neurologist outpatient visits	0.07				0.28					
Inpatient stays	0.01				0.03					
Accident & Emergency visits	0.02				0.56					
Physiotherapy visits	0.13				0.52					
Speech and language therapy visits	0.66				0.11					
Occupational health therapy visits	0.04				0.46					
Other consultant specialist visits	0.09				0.44					
Total cost	0.02				<0.01					

† Test for significant differences in mean values between non-SAC and SAC groups (unadjusted)

‡ Test for significant differences in mean values between non-SAC and SAC groups (adjusted for age, sex and comorbidities)

§ Test for significant differences in mean values by number of symptoms separately for non-SAC and SAC groups (adjusted for age, sex and comorbidities)

SAC, specialist ataxia centre; N, number of participants who responded to that question.

Supplementary Table 12: Time taken to travel to primary ataxia treatment centre

**Duration**	**Patients who reported attending a SAC currently (N=72)**	**Patients who reported never attending a SAC (N=128)**
Less than 1 hour	12 (17%)	80 (63%)
1-2 hours	42 (58%)	27 (21%)
3-4 hours	11 (15%)	2 (2%)
More than 4 hours	0 (0%)	2 (2%)
Did not answer	7 (10%)	17 (13%)
