# Supplementary material for: Comparison of specialist ataxia centres with non-specialist services in terms of treatment, care, health services resource utilisation and costs in the UK using patient-reported data
Source: BMJ Open. 2024 Sep 5;14(9):e084865. doi: 10.1136/bmjopen-2024-084865 (PMC11381710; doi:10.1136/bmjopen-2024-084865)
Supplement: Supplementary file 2 [file bmjopen-2024-084865.reviewer_comments.pdf]

PEER REVIEW HISTORY

BMJ Open publishes all reviews undertaken for accepted manuscripts. Reviewers are asked to complete a checklist review form and are provided with free text boxes to elaborate on their assessment. These free text comments are reproduced below.

ARTICLE DETAILS

**TITLE (PROVISIONAL)**	Comparison of specialist ataxia centres with non-specialist services in terms of treatment, care, health services resource utilisation and costs in the UK using patient reported data.
**AUTHORS**	Vallortigara, Julie; Greenfield, Julie; Hunt, Barry; Hoffman, Deborah; Booth, Suzanne; Morris, Stephen; Giunti, P

VERSION 1 - REVIEW

**REVIEWER NAME**	Watkins, Janette M.
**REVIEWER AFFILIATION**	Indiana University Bloomington, Department of Kinesiology
**REVIEWER CONFLICT OF INTEREST**	I have none to declare
**DATE REVIEW RETURNED**	12-Feb-2024

**GENERAL COMMENTS**	The manuscript addresses an important issue with a survey approach. The results are meaningful and could help guide future research in this area, particularly for increasing patient care and outcomes.

**REVIEWER NAME**	Bunn, Lisa
**REVIEWER AFFILIATION**	University of Plymouth
**REVIEWER CONFLICT OF INTEREST**	nad
**DATE REVIEW RETURNED**	23-Feb-2024

**GENERAL COMMENTS**	This is a specialist area of practice where the provision of specialist versus local neurology management is often debated. Travel related fatigue versus the benefits gained from a specialist team. This is an accessible read and one which provides justification for a model of care for this rare set of diseases.

**REVIEWER NAME**	Stephen, Christopher D.
**REVIEWER AFFILIATION**	Harvard University
**REVIEWER CONFLICT OF INTEREST**	None.
**DATE REVIEW RETURNED**	21-Apr-2024

**GENERAL COMMENTS**	Jaques and colleagues described a survey of 277 UK ataxia patients with a view to assessing the potential benefits of specialist

	ataxia centres (SACs), comparing care pathways, healthcare utilisation and associated costs regarding patients assessing SACs vs. non-specialist centres. They showed higher satisfaction in patients attending SAC, related to a better understanding of their diagnosis. Coordinating referrals and opportunities for participating in research, subjectively better symptom management and more personalized care, without a significant difference in costs associated with attending an SAC. Their formulation was that given the higher patient satisfaction (with similar costs) in patients attending SACs, suggesting that ataxia patients may be better served at SACs, as opposed to centres without similar experience.<br><br>This data is important but is not presented in the most effective or efficient manner and needs to be heavily rewritten and, likely in some areas, re-conceptualised, with substantial concerns from this reviewer regarding many aspects, from writing to methodology. In general, the paper should be made much more concise, both with respect to the article word count and also to the substantial number of tables, almost all of which are redundant, or do not say more than is addressed in the text, or could be addressed in a sentence.<br><br>General comments:<br>- Proofreading is needed, as there are several grammatical errors.<br>- A critical issue is the lack of a clear and concise definition of ataxia, the lack of clarity regarding the disease population (a large proportion of “idiopathic” ataxia) and the considerable heterogeneity, both with respect to diagnosis and the individual carrying out the survey (patients vs. non-patients)<br>- There are far too many tables, often not saying very much<br>- Graphs describing some of the data could also be useful<br>- Much of the results section needs to be completely re-written<br>- The groups YES to SAC and No to SAC is unwieldy and would be better as being SAC vs non-SAC or something similar<br><br><br>Title:<br>“The value of specialist centres in the UK for the management of the ataxias as an example of rare diseases and beyond”. I assume this was supposed to be “an example FOR rare diseases and beyond”? Otherwise, this title does not make sense.<br><br><br>Abstract:<br>- Although implied by the prefix “progressive ataxias”, it may be more accurate to instead describe “cerebellar ataxia” as the patient group excludes other forms e.g. vestibular, sensory and functional ataxias.<br>- The numbers of SAC vs. non-SAC centres and the proportion of the patients attending each of the 2 centres should be included for the reader and to put the data and comparisons in context.<br><br><br>Introduction:<br>- The first paragraph should be re-written to be consistent and more precise in its description of ataxia and be more clear and concise regarding the overall definition of ataxia, which is not clearly stated. I assume this means genetic ataxias? Otherwise, the description does not fit, as ataxia meaning incoordination of any cause, is certainly not rare.

	. “Ataxia is an umbrella term for a group of complex chronic rare neurological disorders affecting<br>motor coordination”. This is not accurate and should be rephrased, as many ataxias are not chronic (such as post-stroke, congenital etc.) and that ataxia as a symptom is not rare at all unless described in purely cerebellar terms (vs. other forms). Later “cerebellar ataxia” is used, which is more precise but not included in the definition here.<br>. “People with ataxia experience lack of balance, as well as impaired movement, slurred speech and impaired eye movements, tremor and difficulty in swallowing, among other symptoms”. I would rephrase this either in formal diagnostic terms (cardinal motor feature soif the cerebellar syndrome), or symptoms, as many patients with ataxia do not have symptoms associatred with their oculomotor disorders (although some can iif they have movements at rest or significant nystagmus, or slowing)<br>. “Other symptoms are variably associated with specific ataxia conditions, including spasticity, sensory disturbance, auditory and visual impairment, bladder and bowel dysfunction and cognitive impairment.” This is again mixing symptoms with signs/clinical decsriptors, as “spasticity” is not a ssymptom (vs. “stiffness”)<br>. “In general, the level of disability increases, resulting in a loss of functional independence” - This is implied by the previous sentence.<br><br>- Second paragraph<br>. “progressive ataxias” needs to be adequately and precisely described and may indicate the genetic inheritance patterns<br>. “Epidemiological evidence is lacking, but estimates suggest that there are at least 10,000 adults and 500 children with progressive ataxia in the UK” – more recent epidemiological data should be cited, as these are quite old and also with a more all-inclusive reference for incidence rates<br><br>- Third paragraph<br>. “A lack of awareness and understanding of these rare neurological disorders among health care professionals makes their management challenging and suggests an important diagnostic and treatment role for specialist clinical services” – references 8 and 9 appear very similar and would benefit from substituting a reference from a different group<br>“with these complex and complex conditions” missing word<br>- Proofread the rest of the paragraph<br><br><br>Methods:<br>- Patients – “all patients (or carers of patients, as proxy) with ataxia” – This all-comers definition without inclusion/exclusion other than age, results in a very heterogeneous population, not all of which would necessarily be “progressive ataxia” or even cerebellar ataxia (although the latter is suggested by Table 1. This could also include non-cerebellar ataxia patients, including FND patients and others, unless specific genes are known or, e.g. if they have VUSs that may not be related to their underlying condition. In addition, including caregivers/family members as proxies would make specific comparisons to patients themselves inaccurate and hence should be compared separately.<br>- Data analyses - Most readers will not necessarily read the survey and hence rather than referring to only question numbers, the text of the question should be stated<br>- The examples of symptoms in question 43 are very broad categories and there are significant omissions (e.g.

	endocrinological, which is very relevant, particularly in the most common ataxia, FA). Also, a description of complexity only based on the number of symptom categories does not necessarily reflect the level of complexity, as this would be based on the severity of each of these symptom categories (which has been attempted, as whether patients were referred for treatment, as opposed to being treated by their GP). If even one of these were severe, this would immediately result in high complexity, as opposed to many mild systemic symptoms. Malignancy (pertinent for AT) is also not included in a main category here and would clearly cause high complexity. Question 9 may, therefore, be more important for this. As an aside, it is unclear why "hemiplegia" is included in question 9, as one-sided symptoms tend not to be a symptom of ataxia (AHC has this but this is episodic and mainly related to dystonia). Also, "stroke" tends to be used as opposed to cerebrovascular accident (which is these days an archaic term).<br>- Resource use and cost - "the wording in the questionnaire could have been misunderstood, as respondents were asked to report the number of visits to the SAC, and this may have been interpreted by respondents to mean the number of visits to see a specialist about their ataxia at the hospital where the SAC was, irrespective of the type of specialist seen". This should be in the discussion and is confusing and should be rephrased.<br>. "All costs were reported in 2021 UK£" - If the survey was conducted in 2019, why was this expressed in 2021 pounds? This is particularly relevant given inflation.<br><br>Results:<br>- Cohort demographics:<br>"With regards to the diagnosis, 114 (42.7%) respondents reported idiopathic cerebellar ataxia (CA), 78 (29.2%) inherited CA, 38 (14.2%) with other types of ataxia, 27 (10.1%) with Friedreich's ataxia (FRDA) and 10 (3.8%) with unknown type". This statement is concerning, as the large proportion of idiopathic ataxia is unusual. It is also concerning as to the accuracy of this very large group, as whether the idiopathic ataxias are truly idiopathic – ILOCA as an entity is very uncommon and unless these are all MSA-C, it would be very important to know more about this idiopathic subgroup. It may not be possible to ascertain this, given the nature of the questionnaire, but it should absolutely be highlighted as a limitation. This has implications for the entire paper.<br>- Why is FA not included in the inherited ataxia group and as a separate group on its own? In the same way, including a group of the SCAs would be important but explicitly stated as subcategories of inherited ataxias.<br><br>Getting a diagnosis:<br>- This title should be rephrased as something like "Reaching a diagnosis" as the current wording is too colloquial.<br>- This needs to be rewritten as needed for grammar, e.g. "Respondents were asked how long ago they were given the diagnosis of ataxia by a healthcare provider" – is this their time from diagnosis and should it be concisely stated?<br>- Time to referral – an immediate referral seems impossible, particularly given that the patients would then essentially have to have communicated with a GP and abruptly referred to neurology which does not seem feasible. The main implications should be more concisely summarised.<br>- That over 5 years is the highest category is problematic. It would have been better asking the patients how long they had symptoms

	of ataxia (disease duration) and separately how many years from symptoms to diagnosis as a number, as opposed to categories.<br><br>Attendance at a SAC:<br>- It is very important that this should be broken down as the following: attendance at a specialist ataxia clinic vs. movement disorders clinic vs. general neurology clinic. This is because movement disorders specialists would have much greater familiarity and ability to manage ataxia patients.<br>- This entire section needs to be far more concisely summarised.<br>- “When we looked at comparing the complexity of the ataxia between the groups YES to SAC and NO to SAC …” This is of concern as it otherwise appears that more complex patients are not being seen at the SAC, as these tend to be mainly treated outside SACs.<br><br>Patient pathway:<br>- That the closing of 1 of the 3 SACs is a big issue, as this was no longer attending not out of choice from the participants<br>- Location should be included in the demographic section. That there are 9 regions of England included and the entire countries of Scotland, Northern Ireland and Wales as single regions is concerning, particularly given the geographic size of these regions. There should be discussion regarding those in Scotland and Northern Ireland in particular, as these are very remote from the SACs.<br><br>Patient satisfaction:<br>- Experience at SAC should be directly compared to non-SAC. There are many sections that are unclear and should be revised.<br><br>Resource use and costs:<br>- “The mean total cost per patient over a one-year period …” The methodology for this should be more clearly described in the methods and the source of estimated information. It seems implausible that an accurate cost could be devised for each patient, particularly as the duration of any appointments (e.g. double appointments for more complex patients) and given that raw numbers of appointments are not described but ranges (other than 1). We also do not know the distance travelled to appointments for each participant or other direct costs, including other appointments, as well as those unrelated to their ataxia. The inpatient and emergency department stays are typically a very large component of costs and it seems impossible for these to have been accounted for (given the lack of testing and length of stay for each presentation), adding further credence to the costs described not being accurate. There is also no discussion of the work-up/investigations, which would be another important facet of this.<br>- This section is also hard to follow and should be re-written if included in a revised version. Given the concerns in the point above, this section may not have the veracity to be included.<br><br>Discussion:<br>- “A high proportion of people (46.5%) did not have a definite diagnosis. Progressive ataxias are frequently a very challenging diagnostic, and even with the use of whole genome sequencing many cases remain undiagnosed.” Data from our centre would refute this statement.<br>- The above limitations I have intimated should also be included

- Not ascertaining whether patients see movement disorder is very pertinent. Given that there are currently only 2 SACs in England, it, therefore, suggests that education and training on ataxia should be given to general and particularly movement disorders specialists to provide better ataxia care. In addition, identifying local PT, OT, and SLPs with an interest and experience in ataxia (particularly those associated with large tertiary referral centres) who could be local champions in areas without SACs would be vital.
- The fact that the SACs are only in England is a major disparity for those outside of England and particularly Northern Ireland and Scotland
- The major concerns regarding the cost data should be highlighted and likely preclude in inclusion
- "The age range of our respondent cohort was somehow unusual for this kind of conditions (a majority were 60-80 years old)" – I agree. This is a major concern, as most patients with hereditary ataxia (other than SCA6 and other particularly late-onset ataxias would be dead by this stage.

Table 1:
- Gender should be sex.
- Why is FA separate from inherited ataxia? Any breakdown in the idiopathic ataxia group will be very important (if not majority MSA-C this is concerning, as idiopathic excludes acquired ataxia causes).
- This must also include geographic region, as this is very important.
- "Attendance to SAC" – the wording is very colloquial in the category breakdown.
- A list and proportion of the genetic ataxias should be included in the caption or otherwise in a supplement

Table 2:
- This is entirely redundant and can be (and is) described in a sentence or could be included in Table 1.

Table 3:
- Similarly, this is redundant and can be described in a sentence, or could be included in Table 1.

Table 4:
- The "Other primary HCP" groups should be defined

Table 5:
- Redundant. Can be described in a sentence or combined into a larger table.

Table 6:
- As above.

Table 7:
- Very confusing and should be re-written for clarity.

Table 8:
- As above, likely redundant.

Table 9:
- Only need positive for each question, as the negative is just the remaining % from 100%

Supplementary Figure 1:

	- Too many colours and difficult to distinguish between groups

**REVIEWER NAME**	Ibrahim, Norlinah Mohamed
**REVIEWER AFFILIATION**	UKM, Department of Medicine
**REVIEWER CONFLICT OF INTEREST**	None
**DATE REVIEW RETURNED**	02-May-2024

**GENERAL COMMENTS**	This is a well conducted survey-based study, as part of a larger study in Europe to determine patient-reported outcomes related ataxia care in the UK and costs related to the care. The paper is clearly written, although very lengthy. Here are some suggestions to improve the readability<br>1. How many respondents were there. Table 1 mentions 273, while the manuscript states 277.<br>2. Suggest to add in the manuscript the n and % of those in the Yes, No and Used to group.<br>3. More than 50% of patients were in the No to SAC category. Perhaps this can be explained in the discussion e.g. due to access or preference of patients. The unequal number from the Yes and No categories which would affect the findings. This should be included in the limitation<br>4. The paper is very lengthy and some results such as feedback can be presented in table format and not repeated in sentences.<br>5. There are too many tables. Tables can be merged together.<br>6. Suggest to discuss on why patients who attended SAC had negative feedback on help to get benefits. This would be an important aspect to look into.<br>7. Table 9 doesn't include No to SAC. Table 10 - is referring to positive feedback. Perhaps can elaborate what is meant by positive feedback.<br>8. There is percentage of total respondent for each table which is not required, as it should total to 100%

VERSION 1 – AUTHOR RESPONSE

Reviewer 3 Comments:

General comments:

• Proofreading is needed, as there are several grammatical errors. We apologise for submitting the previous version with minimum proofreading, and we have now proofread the paper to ensure there were no grammatical errors.

• A critical issue is the lack of a clear and concise definition of ataxia, the lack of clarity regarding the disease population (a large proportion of “idiopathic” ataxia) and the considerable heterogeneity, both with respect to diagnosis and the individual carrying out the survey (patients vs. non-patients). We have made some changes in the introduction in order to address this point.

• There are far too many tables, often not saying very much. We have removed 5 tables.

• Graphs describing some of the data could also be useful. We have decided to keep data presented as tables as we felt that the data was clearer presented in table format.

• Much of the results section needs to be completely re-written. The results section has been revised together with removing some tables.

• The groups YES to SAC and No to SAC is unwieldy and would be better as being SAC vs non-SAC or something similar. We have now used SAC and non-SAC throughout the whole manuscript.

• Title:

• "The value of specialist centres in the UK for the management of the ataxias as an example of rare diseases and beyond". I assume this was supposed to be "an example FOR rare diseases and beyond"? Otherwise, this title does not make sense. We have changed the title of the manuscript to 'Comparison of specialist ataxia centre service with non-specialist service in terms of treatment, care, health services resource utilisation and costs in the UK using patient reported data'.

• Abstract:

• Although implied by the prefix "progressive ataxias", it may be more accurate to instead describe "cerebellar ataxia" as the patient group excludes other forms e.g. vestibular, sensory and functional ataxias. We have amended this sentence.

• The numbers of SAC vs. non-SAC centres and the proportion of the patients attending each of the 2 centres should be included for the reader and to put the data and comparisons in context. The number of SAC is mentioned in the methods section. We refer as non-SAC centres the standard neurology clinics that people can access locally. We do not have access to the number of such clinics. Because we did not ask which centre people attended for their ataxia, we are not able to provide information on the proportion of people who attended the London and Sheffield centres.

• Introduction:

• The first paragraph should be re-written to be consistent and more precise in its description of ataxia and be more clear and concise regarding the overall definition of ataxia, which is not clearly stated. I assume this means genetic ataxias? Otherwise, the description does not fit, as ataxia meaning incoordination of any cause, is certainly not rare. The majority of patients with ataxia who are members of Ataxia UK are suffering with a neurodegenerative disorder of which some are genetically determined. We have made some changes to this section of the introduction to make the definition of the ataxias clearer to the readers. The conditions covered for this study are in line with those described in the Medical Guidelines for the management of the ataxias published by Ataxia UK on page 4 and 'exclude disorders where ataxia is an epiphenomenon of another neurological condition (see Table 1a and 1b). ' https://www.ataxia.org.uk/healthcare-professionals/resources-for-healthcare-professionals/medical-guidelines/introduction-and-full-document/

• "Ataxia is an umbrella term for a group of complex chronic rare neurological disorders affecting motor coordination". This is not accurate and should be rephrased, as many ataxias are not chronic (such as post-stroke, congenital etc.) and that ataxia as a symptom is not rare at all unless described in purely cerebellar terms (vs. other forms). Later "cerebellar ataxia" is used, which is more precise but not included in the definition here. We have amended the wording to clarify the population included in this study. The ataxia patients who are attending the specialist ataxia centres and who are members of Ataxia UK are patients with progressive neurodegenerative disorders.

• "People with ataxia experience lack of balance, as well as impaired movement, slurred speech and impaired eye movements, tremor and difficulty in swallowing, among other symptoms". I would rephrase this either in formal diagnostic terms (cardinal motor feature so if the cerebellar syndrome), or symptoms, as many patients with ataxia do not have symptoms associatred with their oculomotor disorders (although some can iif they have movements at rest or significant nystagmus, or slowing). We have changed that sentence to "People with ataxia experience lack of balance, variably

associated with slurred speech, double vision, tremor and difficulty in swallowing, among other symptoms."

• "Other symptoms are variably associated with specific ataxia conditions, including spasticity, sensory disturbance, auditory and visual impairment, bladder and bowel dysfunction and cognitive impairment." This is again mixing symptoms with signs/clinical decsriptors, as "spasticity" is not a symptom (vs. "stiffness"). We have re-written that section of the introduction to address this point and mentioned symptoms in a clearer and more organized manner.

• "In general, the level of disability increases, resulting in a loss of functional independence" - This is implied by the previous sentence. We have removed this sentence.

• Second paragraph

• "progressive ataxias" needs to be adequately and precisely described and may indicate the genetic inheritance patterns. We have changed that sentence to "The ataxias are a group of conditions that are clinically and genetically heterogenous."

• "Epidemiological evidence is lacking, but estimates suggest that there are at least 10,000 adults and 500 children with progressive ataxia in the UK" – more recent epidemiological data should be cited, as these are quite old and also with a more all-inclusive reference for incidence rates. There is not more recent epidemiology study data in the UK.

• Third paragraph

• "A lack of awareness and understanding of these rare neurological disorders among health care professionals makes their management challenging and suggests an important diagnostic and treatment role for specialist clinical services" – references 8 and 9 appear very similar and would benefit from substituting a reference from a different group. We have added two more references.

• "with these complex and complex conditions" missing word. We have added the missing word.

• Proofread the rest of the paragraph. We have proofread the whole revised manuscript.

• Methods:

• Patients – "all patients (or carers of patients, as proxy) with ataxia" – This all-comers definition without inclusion/exclusion other than age, results in a very heterogeneous population, not all of which would necessarily be "progressive ataxia" or even cerebellar ataxia (although the latter is suggested by Table 1. This could also include non-cerebellar ataxia patients, including FND patients and others, unless specific genes are known or, e.g. if they have VUSs that may not be related to their underlying condition. The inclusion/exclusion criteria were age, location and having a diagnosis of ataxia. We wanted in this survey to include all types of ataxias that participants may live with and based on what people reported in terms of diagnosis, the types are listed in Table 1. In that table, we have presented the results of reported diagnosis in five categories as it was designed in the survey, and we added additional ataxia types in the comments below the table as suggested by the patients who filled the forms and wrote in free text boxes.

• In addition, including caregivers/family members as proxies would make specific comparisons to patients themselves inaccurate and hence should be compared separately. It was clearly instructed to the caregiver and family to support patients if needed in filling in the questionnaire, and that the patient would be consulted for the questions to answer. It was written in page 1 of the survey presented in Appendix 1: "Parents/carers/guardians filling in the survey should respond as per the patient's opinion, not your own.". So, the questionnaires filled by caregivers or family members would be representative of the patients' voices, experience and feedback. Therefore, we have considered

these responses together and dealt in this paper with one group of respondents. In no circumstances we have had contradictory responses in surveys filled by patients compared to those filled by another person on behalf of the patient.

• Data analyses - Most readers will not necessarily read the survey and hence rather than referring to only question numbers, the text of the question should be stated. We have decided not to include the text of the questions as this would make the section of material and methods even more lengthy. The survey is accessible in Appendix 1 for reference to questions mentioned.

• The examples of symptoms in question 43 are very broad categories and there are significant omissions (e.g. endocrinological, which is very relevant, particularly in the most common ataxia, FA). Also, a description of complexity only based on the number of symptom categories does not necessarily reflect the level of complexity, as this would be based on the severity of each of these symptom categories (which has been attempted, as whether patients were referred for treatment, as opposed to being treated by their GP). If even one of these were severe, this would immediately result in high complexity, as opposed to many mild systemic symptoms. Malignancy (pertinent for AT) is also not included in a main category here and would clearly cause high complexity. Question 9 may, therefore, be more important for this. As an aside, it is unclear why “hemiplegia” is included in question 9, as one-sided symptoms tend not to be a symptom of ataxia (AHC has this but this is episodic and mainly related to dystonia). Also, “stroke” tends to be used as opposed to cerebrovascular accident (which is these days an archaic term). AT is not included in this study as patients living with this type of ataxia are supported by another charity in the UK and therefore would not have been asked to complete the survey. Question 9 was asked to know about any co-morbidities that people might live with, this is why hemiplegia is in that list. To refer to the complexity of ataxia, we listed in question 43 the main symptoms associated with ataxia, without being exhaustive in that list and people could add another symptom in the text box below the question. We agree that if one additional symptom is really severe, this would immediately result in the driver for the severity of the disease. However, this is extremely rare in patients with these conditions.

• Resource use and cost - “the wording in the questionnaire could have been misunderstood, as respondents were asked to report the number of visits to the SAC, and this may have been interpreted by respondents to mean the number of visits to see a specialist about their ataxia at the hospital where the SAC was, irrespective of the type of specialist seen”. This should be in the discussion and is confusing and should be rephrased. We have moved the details of this point to the limitations in the Discussion section and rephrased the wording to avoid confusion.

• “All costs were reported in 2021 UK£” - If the survey was conducted in 2019, why was this expressed in 2021 pounds? This is particularly relevant given inflation. Costs are reported in 2021 prices as this was the current/most recent year available at the time of analysis. It was appropriate to use the most up-to-date figures precisely to account for inflation. This is recommended practice for cost analyses in the UK. See for example: https://www.nice.org.uk/process/pmg36/chapter/economic-evaluation-2#evidence-on-resource-use-and-costs.

• Results:

• Cohort demographics:

• “With regards to the diagnosis, 114 (42.7%) respondents reported idiopathic cerebellar ataxia (CA), 78 (29.2%) inherited CA, 38 (14.2%) with other types of ataxia, 27 (10.1%) with Friedreich’s ataxia (FRDA) and 10 (3.8%) with unknown type”. This statement is concerning, as the large proportion of idiopathic ataxia is unusual. It is also concerning as to the accuracy of this very large group, as whether the idiopathic ataxias are truly idiopathic – ILOCA as an entity is very uncommon and unless these are all MSA-C, it would be very important to know more about this idiopathic subgroup. It may not be possible to ascertain this, given the nature of the questionnaire, but it should absolutely be

highlighted as a limitation. This has implications for the entire paper. The diagnoses proposed in the questionnaire were based on the conditions that Ataxia UK members reported to live with. We were looking with the survey at including the majority of the members of Ataxia UK in terms of diagnosis. Therefore, these categories are what were available in the questionnaire. In addition, this is not reflective of the proportion of the population in the UK but of the people who responded to the survey.

• Why is FA not included in the inherited ataxia group and as a separate group on its own? In the same way, including a group of the SCAs would be important but explicitly stated as subcategories of inherited ataxias. Among the inherited ataxias, FA has always been the most numerous. FA is the most complex condition with multiple needs beyond neurology, therefore we wanted to target specifically this group to understand if these very complex patients prefer going to SAC rather than the general neurology clinic. We would like to highlight that the survey was designed with patients in mind and their substantial input rather than from the perspective of healthcare professionals.

• Getting a diagnosis:

• This title should be rephrased as something like "Reaching a diagnosis" as the current wording is too colloquial. We have changed the title to 'Reaching a diagnosis.'

• This needs to be rewritten as needed for grammar, e.g. "Respondents were asked how long ago they were given the diagnosis of ataxia by a healthcare provider" – is this their time from diagnosis and should it be concisely stated? We have rewritten this sentence.

• - Time to referral – an immediate referral seems impossible, particularly given that the patients would then essentially have to have communicated with a GP and abruptly referred to neurology which does not seem feasible. The main implications should be more concisely summarised. This sentence has been rephrased.

• That over 5 years is the highest category is problematic. It would have been better asking the patients how long they had symptoms of ataxia (disease duration) and separately how many years from symptoms to diagnosis as a number, as opposed to categories. This is a good comment. We retrospectively also thought this, and it would have been better to ask people a precise date for these data in order to address that point.

• Attendance at a SAC:

• It is very important that this should be broken down as the following: attendance at a specialist ataxia clinic vs. movement disorders clinic vs. general neurology clinic. This is because movement disorders specialists would have much greater familiarity and ability to manage ataxia patients. This was not possible to do that as this specific question was not posed. In the UK, Ataxia patients have been followed up by a mix of services, the majority being general neurologist and movement disorders services.

• This entire section needs to be far more concisely summarised. This section was reviewed and shortened.

• "When we looked at comparing the complexity of the ataxia between the groups YES to SAC and NO to SAC …" This is of concern as it otherwise appears that more complex patients are not being seen at the SAC, as these tend to be mainly treated outside SACs. This result is addressed in the discussion. There are challenges for more complexes cases to use SAC – the barriers to access a SAC in this paper (travelling) can be a significant burden particularly for those who experience many different symptoms associated with ataxia. This is not surprising to us that as the complexity of the ataxia increases, people might go less to a SAC. As the disease progresses it can become difficult to reach the centre, particularly in the UK which had at the time of the survey two centres open and running.

• Patient pathway:

• That the closing of 1 of the 3 SACs is a big issue, as this was no longer attending not out of choice from the participants. Yes, we could not agree more. We are pleased to say that since this study, a third centre has been accredited by Ataxia UK, it's running and seeing patients. This is stated in the discussion.

• Location should be included in the demographic section. That there are 9 regions of England included and the entire countries of Scotland, Northern Ireland and Wales as single regions is concerning, particularly given the geographic size of these regions. There should be discussion regarding those in Scotland and Northern Ireland in particular, as these are very remote from the SACs. We have presented the living location of the participants in supplementary figures 1 and 2. We have added these results to the demographic section. We have added a sentence about the discussion about the challenge for people in Scotland and Northen Ireland when we discussed the difficulty to access centres.

• Patient satisfaction:

• Experience at SAC should be directly compared to non-SAC. There are many sections that are unclear and should be revised. We have explained in the text the comparisons between groups and the use of the third group of participants who used to go to a SAC and have stopped going.

• Resource use and costs:

• "The mean total cost per patient over a one-year period …" The methodology for this should be more clearly described in the methods and the source of estimated information. It seems implausible that an accurate cost could be devised for each patient, particularly as the duration of any appointments (e.g. double appointments for more complex patients). Patients were asked to record the numbers of different types of contact they had, and we have no reason to believe these would be inaccurate. We did not ask patients to record the duration of each contact, but it is common health economic evaluation methodology to apply unit costs per contact, which is what we have done here (see, e.g., here https://www.nice.org.uk/process/pmg36/chapter/economic-evaluation-2#evidence-on-resource-use-and-costs). This implies that the duration of contacts among patients in the survey is equal to the national average duration across all patients, which we believe is a reasonable assumption.

• and given that raw numbers of appointments are not described but ranges (other than 1). In the Table reporting resource use and costs we report the variation in resource use, as standard deviations, as well as point estimates (as both means and medians).

• We also do not know the distance travelled to appointments for each participant or other direct costs, including other appointments, as well as those unrelated to their ataxia. We measured costs from the perspective of the National Health Service and Personal Social Services, which is recommend methodology in the UK (NICE health technology evaluations: the manual. NICE process and methods [PMG36]Published: 31 January 2022 Last updated: 31 October 2023. 4 Economic evaluation. https://www.nice.org.uk/process/pmg36/chapter/economic-evaluation-2#evidence-on-resource-use-and-coststherefore), as is recommended in UK-based economic analyses we did not include time and travel costs incurred by patients in the cost analysis.

• The inpatient and emergency department stays are typically a very large component of costs and it seems impossible for these to have been accounted for (given the lack of testing and length of stay for each presentation), adding further credence to the costs described not being accurate. As noted, and as reported in the paper, we did include inpatient stays and emergency department visits; survey participants were asked to record the number of these contacts.

• There is also no discussion of the work-up/investigations, which would be another important facet of this. These are included in the unit costs associated with each type of contact; we now state this more clearly in the text.

• This section is also hard to follow and should be re-written if included in a revised version. Given the concerns in the point above, this section may not have the veracity to be included. We have amended the text to make it simpler to understand this results section on the analyses done by comorbidities and number symptoms associated with ataxia. Given that the cost analysis conforms to standard health economic methodology (see, e.g., here https://www.nice.org.uk/process/pmg36/chapter/economic-evaluation-2#evidence-on-resource-use-and-costs), and given that health economic data for people affected by Ataxia are rare we prefer to retain this analysis in the paper.

• Discussion:

• “A high proportion of people (46.5%) did not have a definite diagnosis. Progressive ataxias are frequently a very challenging diagnostic, and even with the use of whole genome sequencing many cases remain undiagnosed.” Data from our centre would refute this statement.

• The above limitations I have intimated should also be included. We have added a sentence in the discussion to set the expectations about how the data collection and analysis were done based on the fact that this survey design was patient rather than HCP driven, aiming to capture ataxia patients views, experience and feedback. Experience from different centres can show that the proportion of people without a definite diagnosis can vary, and with this study it also depends on who has responded to the survey.

• Not ascertaining whether patients see movement disorder is very pertinent. Given that there are currently only 2 SACs in England, it, therefore, suggests that education and training on ataxia should be given to general and particularly movement disorders specialists to provide better ataxia care. In addition, identifying local PT, OT, and SLPs with an interest and experience in ataxia (particularly those associated with large tertiary referral centres) who could be local champions in areas without SACs would be vital. We agree and we state that in the discussion.

• The fact that the SACs are only in England is a major disparity for those outside of England and particularly Northern Ireland and Scotland. We have added a sentence about that point in the discussion

• The major concerns regarding the cost data should be highlighted and likely preclude in inclusion As indicated above, we prefer to retain the analysis of the cost data.

• “The age range of our respondent cohort was somehow unusual for this kind of conditions (a majority were 60-80 years old)” – I agree. This is a major concern, as most patients with hereditary ataxia (other than SCA6 and other particularly late-onset ataxias would be dead by this stage. This age range represents the people who responded to the survey and not the whole population living with ataxia in the UK. This point is mentioned in the discussion, in the limitations of the survey section.

• Table 1:

• Gender should be sex. We have changed that in the table.

• Why is FA separate from inherited ataxia? Any breakdown in the idiopathic ataxia group will be very important (if not majority MSA-C this is concerning, as idiopathic excludes acquired ataxia causes). As previously said, in that table, we have presented the results of reported diagnosis in five categories as it was designed in the survey, and we added additional ataxia types in the comments below the table as suggested by the patients who filled the forms and wrote in free text boxes. The diagnoses

proposed in the questionnaire were based on the conditions that Ataxia UK members reported to live with. We were looking with the survey at including the majority of the members of Ataxia UK in terms of diagnosis. Therefore, these categories are what were available in the questionnaire. Among the inherited ataxias, FA has always been the most numerous. FA is the most complex condition with multiple needs beyond neurology, therefore we wanted to target specifically this group to understand if these very complex patients prefer going to SAC rather than the general neurology clinic. We would like to highlight that the design of the survey has been patient driven rather than neurogeneticist or other healthcare professional driven. We do not have data to give a breakdown of the idiopathic ataxia group.

• This must also include geographic region, as this is very important. The living location is available in supplementary data figure 1 and 2 and is added to the results section.

• “Attendance to SAC” – the wording is very colloquial in the category breakdown. We have changed the wording to SAC, non-SAC and Used to SAC for the groups.

• A list and proportion of the genetic ataxias should be included in the caption or otherwise in a supplement. This is not available as we did not ask for the exact genetic type from participants. Some respondents put their genetic diagnosis result in the text box but not all respondents did therefore we are not able to provide an exhaustive list and proportion of the genetic ataxias.

• Table 2:

• This is entirely redundant and can be (and is) described in a sentence or could be included in Table 1. We agree – this table is removed, and we added data in the text.

• Table 3:

• Similarly, this is redundant and can be described in a sentence, or could be included in Table 1. We agree – this table is removed, and we added data in the text.

• Table 4: this is now Table 2

• The “Other primary HCP” groups should be defined – we have added the definition from the questionnaire in the caption of this table.

• Table 5:

• Redundant. Can be described in a sentence or combined into a larger table. We have removed this table and we added data in the text.

• Table 6:

• As above. We have removed this table and we added data in the text.

• Table 7: this is now Table 3

• Very confusing and should be re-written for clarity. We have changed the title and modified this table and added a sentence to explain how the results are presented: stratified by SAC attendance. This table summarises the responses of access to an MDT whether people are part of SAC, non-SAC or Used to SAC group. We have amended the text in the results section for more clarity.

• Table 8:

• As above, likely redundant. We have removed this Table.

• Table 9: this is now Table 4

• Only need positive for each question, as the negative is just the remaining % from 100%. We understand that comment, however we prefer to have all data, positive and negative presented for clarity and easy read.

• Supplementary Figure 1:

• Too many colours and difficult to distinguish between groups – we have many areas to represent therefore it leads to the use of many different colours.

Reviewer 4 Comments:

• 1. How many respondents were there. Table 1 mentions 273, while the manuscript states 277. As explained in the material and methods about the data cleaning of the survey responses, “incomplete surveys were not removed from the analysis, as respondents chose to answer some questions and to skip others. The number of respondents was reported for each individual question. “This will lead to a different number of respondents from one question to the next.

• 2. Suggest to add in the manuscript the n and % of those in the Yes, No and Used to group. We have added both N and % to the groups throughout the results section.

• 3. More than 50% of patients were in the No to SAC category. Perhaps this can be explained in the discussion e.g. due to access or preference of patients. The unequal number from the Yes and No categories which would affect the findings. This should be included in the limitation. The recruitment of the participants was done through Ataxia UK membership, website, magazine and social media. We did not use the SAC specifically for recruitment of participants. Therefore, this led to these groups SAC and non-SAC based on voluntary participation of patients to this study thanks to the publicity from patient group. The difficulty about accessing a SAC was asked through questions about why people stopped going to a SAC (question number 23e) and why people never went to a SAC (question 23i) and discussed in the discussion. Overall, we have 51.6% of people who have never been to a SAC and 48.4% of people who have been to a SAC (currently attending or used to attend) therefore these two categories are not dissimilar.

• 4. The paper is very lengthy and some results such as feedback can be presented in table format and not repeated in sentences. We have made an attempt to reduce the text, however removing tables can lead to describe the data in the text so more words are needed for that.

• 5. There are too many tables. Tables can be merged together. We have removed five Tables from the results sections.

• 6. Suggest to discuss on why patients who attended SAC had negative feedback on help to get benefits. This would be an important aspect to look into. We explain that feedback from patients by the fact that there is a lack of liaison between SACs and social services. This result is discussed in the discussion with the following sentence: “Aspects of the SAC service that could be improved from the participants perspective are around helping people to access benefits and liaising with social workers. Financial difficulties and lack of access to social services can have a significant impact on quality of life 23. Improving these aspects of the service could potentially facilitate a package of care that is better adapted to their needs. To tackle that gap, someone within the SAC, but also in the charity Ataxia UK could take on that role, or maybe creating a new role as care manager.”

• 7. Table 9 doesn't include No to SAC. The same question wasn’t asked to Non-SAC group. Table 10 - is referring to positive feedback. Perhaps can elaborate what is meant by positive feedback. We have added a sentence in caption of this Table to explain the meaning of positive feedback for clarity.

• 8. There is percentage of total respondent for each table which is not required, as it should total to 100% - Yes that is true.

VERSION 2 - REVIEW

**REVIEWER NAME**	Ibrahim, Norlinah Mohamed
**REVIEWER AFFILIATION**	UKM, Department of Medicine
**REVIEWER CONFLICT OF INTEREST**	None
**DATE REVIEW RETURNED**	24-Jul-2024

**GENERAL COMMENTS**	The authors have addressed the comments and concerns raised. The paper is suitable for publication
